# Pathogen-driven gene expression patterns lead to a novel approach to the identification of common therapeutic targets

**DOI:** 10.1038/s41598-022-25102-8

**Published:** 2022-12-06

**Authors:** Mohammad Uzzal Hossain, Nadim Ferdous, Mahjerin Nasrin Reza, Ishtiaque Ahammad, Zachary Tiernan, Yi Wang, Fergus O’Hanlon, Zijia Wu, Shishir Sarker, A. K. M. Mohiuddin, Keshob Chandra Das, Chaman Ara Keya, Md. Salimullah

**Affiliations:** 1 Department of Pharmacology, Medical Sciences Division, University of Oxford, Oxford, OX13QT UK; 2 Bioinformatics Division, National Institute of Biotechnology, Ganakbari, Ashulia, Savar, Dhaka, 1349 Bangladesh; 3 Department of Biotechnology and Genetic Engineering, Mawlana Bhashani Science and Technology University, Santosh, Tangail, 1902 Bangladesh; 4 Mathematical Institute, University of Oxford, Oxford, OX2 6GG UK; 5 Department of Chemistry, University of Oxford, Oxford, OX2 6GG UK; 6 Department of Microbiology, Jagannath University, Dhaka, 1100 Bangladesh; 7 Molecular Biotechnology Division, Ministry of Science and Technology, National Institute of Biotechnology, Ganakbari, Ashulia, Savar, Dhaka, 1349 Bangladesh; 8 Department of Biochemistry and Microbiology, North South University, Dhaka, 1229 Bangladesh

**Keywords:** Computational biology and bioinformatics, Microbiology, Structural biology

## Abstract

Developing a common medication strategy for disease control and management could be greatly beneficial. Investigating the differences between diseased and healthy states using differentially expressed genes aids in understanding disease pathophysiology and enables the exploration of protein-drug interactions. This study aimed to find the most common genes in diarrhea-causing bacteria such as *Salmonella enterica* serovar Typhimurium, *Campylobacter jejuni*, *Escherichia coli*, *Shigella dysenteriae* (CESS) to find new drugs. Thus, differential gene expression datasets of CESS were screened through computational algorithms and programming. Subsequently, hub and common genes were prioritized from the analysis of extensive protein–protein interactions. Binding predictions were performed to identify the common potential therapeutic targets of CESS. We identified a total of 827 dysregulated genes that are highly linked to CESS. Notably, no common gene interaction was found among all CESS bacteria, but we identified 3 common genes in both *Salmonella-Escherichia* and *Escherichia-Campylobacter* infections. Later, out of 73 protein complexes, molecular simulations confirmed 5 therapeutic candidates from the CESS. We have developed a new pipeline for identifying therapeutic targets for a common medication strategy against CESS. However, further wet-lab validation is needed to confirm their effectiveness**.**

## Introduction

Microarrays have revolutionized biotechnology, allowing researchers to track down the expression of tens of thousands of genes simultaneously^1^. In most cases, any microarray experiment results in a list of genes found to be differentially expressed. The analysis of these large-scale gene expressions has become a fundamental approach to the identification of clinical diagnostic factors as well as potential drug targets^2^. The common challenge here is translating such lists of gene expression data into a better understanding of the underlying disease phenomena. The first solution in this direction can be to translate the gene expression pattern into a functional profile, which will offer insight into the cellular mechanisms relevant to the given disease condition^3^. Over the last decade, high-throughput in silico genomics, transcriptomics, and proteomics technologies have allowed researchers to rapidly acquire and analyze several thousand gene expression profiles in any experiment^4^.

Enteric bacterial pathogens and parasites are the leading cause of infectious diarrhea in developing countries^5^. Common bacteria that cause diarrhea include *Salmonella*, *Escherichia*, *Shigella*, and *Campylobacter*. The virulence of *Salmonella enterica* serovar Typhimurium (*S*. Typhimurium) greatly depends on two types III secretion systems (T3SSs) which are encoded in pathogenicity islands 1 (SPI1) and 2 (SPI2), respectively^6^. These systems translocate proteins called effectors into a eukaryotic host cell, where they interfere with certain host signal transduction pathways to allow the internalization of pathogens and their survival and proliferation inside vacuoles^6^. *Escherichia coli (E. coli)* is the primary cause of watery diarrhea in infants, often accompanied along with causing low-grade fever and vomiting^7^. Compared with other pathogens such as *Shigella* and *Salmonella*, *E. coli* is typically considered non-invasive. However, it encodes a T3SS producing a characteristic attaching and effacing (A/E) lesion^8^. The effacement of microvilli on the epithelial surface induced by A/E lesions contributes to the diarrheal phenotype owing to the loss of overall absorptive surface^8^. Out of the four major *Shigella* species that cause diarrheal disease, *Shigella sonnei (S. sonnei)* and *Shigella flexneri (S. flexneri)* are the most common species in the U.S. and other developed countries^9^. However, the changing scenario was observed in a 2013 study that shows the sudden emergence of *S. sonnei* in Bangladesh^10^. The two other *Shigella* species, *Shigella dysenteriae (S. dysenteriae),* and *Shigella boydii (S. boydii)* have a generally low infection rate and are found very rarely in developed countries. *S. dysenteriae* produces Shiga toxin, making it the most life-threatening of all of these infections, which can also lead to hemolytic uremic syndrome (HUS). Releasing the exotoxin *by S. dysenteriae* compromises the central nervous system and the gut, while enterotoxin causes the diarrhea^11^. The primary cause of inflammation by *Shigella* involves various steps of the invasion process. An initial release of IL-1β by the destruction of macrophages after emergence from M-cells attracts polymorphonuclear leukocytes (PMNs) that release a precursor to the secretagogue adenosine, ultimately activating Cl − secretion. The presence of free bacteria on the basolateral side of cells aggravates this early step in inflammation^12,13^. *Campylobacter jejuni (C. jejuni)* initiates infection by penetrating the gastrointestinal mucus using its high motility and spiral shape^14^. Then they adhere to the gut enterocytes and induce diarrhea by toxin release. *C. jejuni* releases several different enterotoxins and cytotoxins varying from strain to strain, and the severity of enteritis correlates with these toxins^14^. These four bacterial infections in the human body have the common disease-causing phenomena which is diarrhea. A significant number of host (human) genes get upregulated and downregulated simultaneously during these pathogenesis events by four diarrhea-causing bacteria. Thus, it is important to decipher the molecular mechanisms underlying these dysregulated gene networks in the pathogenesis of diarrhea.

Previously, several investigations have been conducted individually on the four bacteria to deduce how their gene expression events contribute to various intestinal and systemic infections^15–18^. However, to date, no work has been done to reveal the gene–gene associations and their dysregulation due to the pathogenesis of these four bacteria. Therefore, we have addressed all of these four bacteria in our study and disclosing the significant gene networks of dysregulated genes due to these bacterial infections would reveal plausible drug targets and shed light on the possibility of common therapy.

All the molecular events in the cell are controlled primarily by changes in the expression of key genes. Gene transcription is pivotal in regular events such as cell division, proliferation, differentiation, and cell death. Much interest is therefore focused on depicting gene expression profiles to identify the key gene clusters whose expression is changed in disease states. Gene–gene interactions provide us the information to what extent and how the gene share the relation with the other genes. One gene can interact with other genes by several ways including domain, motif, co-localization, pathways etc. For this reason, one gene can have the great influence on another gene’s function and regulation. Therefore, the goal of this study was to reveal the commonly dysregulated genes and the significant gene networks associated with these four bacterial infections. We have analyzed the gene expression pattern, hub genes identification pathways, regulatory biomarkers and structural associations of interacted proteins involved in these disease progressions. Furthermore, we investigated the functional associations of final products of these dysregulated genes to scrutinize the major drug targets for common therapy.

## Results

### Analysis of microarray data

Four gene expression profiles were used in this investigation, namely (GSE51043, GSE18810, GSE19315, and GSE36701). A total of 215 DEGs (Differentially Expressed Genes) were screened from GSE51043 with 72 upregulated genes and 143 downregulated genes. When the GSE18810 dataset was examined, it yielded 187 DEGs, 50 of which were upregulated and 137 of which were downregulated. In the GSE19315 gene chip, 214 DEGs were discovered, with 109 upregulated genes and 105 downregulated genes. Finally, the GSE36701 dataset yielded 213 DEGs, with 67 upregulated and 146 downregulated genes. All the upregulated and downregulated DEGs resulting from four bacterial pathogenesis are enlisted in Supplementary files 1–2.

### Gene ontologies (GO) and signaling pathways

In a complex disease state, a varying range of signaling pathways and GO terms are involved in the progression of diseases. In this process, we used all the DEGs (298 upregulated and 529 downregulated genes) to determine significant pathways and gene ontologies that may link diarrhea pathogenesis. GO terms and pathways were selected based on the number of genes involved and having a p-value less than or equal to 0.05. Top GO terms were identified as regulation of cell population proliferation (27), RNA polymerase II cis-regulatory region sequence-specific DNA binding (25) in upregulated genes and nervous system development (20), cis-regulatory region sequence-specific DNA binding (39) in downregulated genes. In addition to GO terms, over-presented signaling pathways were predicted for DEGs. The top pathways are cancer pathways (12), cytokine-cytokine receptor interaction (10) in upregulated genes, Chemical carcinogenesis (12), and Rap1 signaling pathway (11) in downregulated genes. Supplementary File 3–5 contains the list of all GO terms and pathways of DEGs based p-value less than or equal to 0.05. Furthermore, we identified the commonly altered pathways (both due to upregulation and downregulation) from the top 30 pathways of each DEG set resulting from the pathogenesis of the four bacteria shown in Table 1.

**Table 1:** Common altered pathways, names of the DEGs and the responsible pathogens for upregulation or downregulation of these DEGs.

Regulation mechanism	Pathways	Genes	Responsible pathogen for altering pathways
Upregulation	Insulin signaling pathway	RPS6, HK2, PPP1CA	*Campylobacter*
		RPS6KB1, IRS1	*Shigella*
	Dopaminergic synapse	CAMK2B, PPP1CA	*Campylobacter*
		GNB5	*E. coli*
	Cytokine-cytokine receptor interaction	CCL5, XCL2, XCL1	*Campylobacter*
		TNFRSF19, GDF3	*E. coli*
	Autophagy	ERN1, PRAP1	*Campylobacter*
		RPS6KB1, IRS1	*Shigella*
	Necroptosis	CAMK2B, FTL	*Campylobacter*
		STAT5B, TNFAIP3	*Salmonella*
	ErbB signaling pathway	JUN, RPS6KB1, HBEGF	*Shigella*
		STAT5B, EREG	*Salmonella*
Downregulation	Steroid biosynthesis	FDFT1	*Campylobacter*
		EBP, DHCR24, DHCR7	*Salmonella*
	Circadian entrainment	NOS1AP, ADCY6	*Campylobacter*
		GNAO1, ADCY2	*Shigella*
	Progesterone-mediated oocyte maturation	RPS6KA3, ADCY6	*Campylobacter*
		MAPK10, ADCY2	*Shigella*
		PIK3R3, PIK3CD, PIK3R1, CPEB4, ADCY5	*Salmonella*
	Circadian rhythm	NPAS2	*Campylobacter*
		RORB	*E. coli*
	Retrograde endocannabinoid signaling	NDUFB4, ADCY6	*Campylobacter*
		GNAO1, MAPK10, NAPEPLD, ADCY2	*Shigella*
	Oxytocin signaling pathway	CACNB2, ADCY6	*Campylobacter*
		GNAO1, ADCY2, EGFR	*Shigella*
	GnRH signaling pathway	GNRH1, ADCY6	*Campylobacter*
		MAPK10, ADCY2, EGFR	*Shigella*

### Common genes identified within DEGs

**Figure 1:**
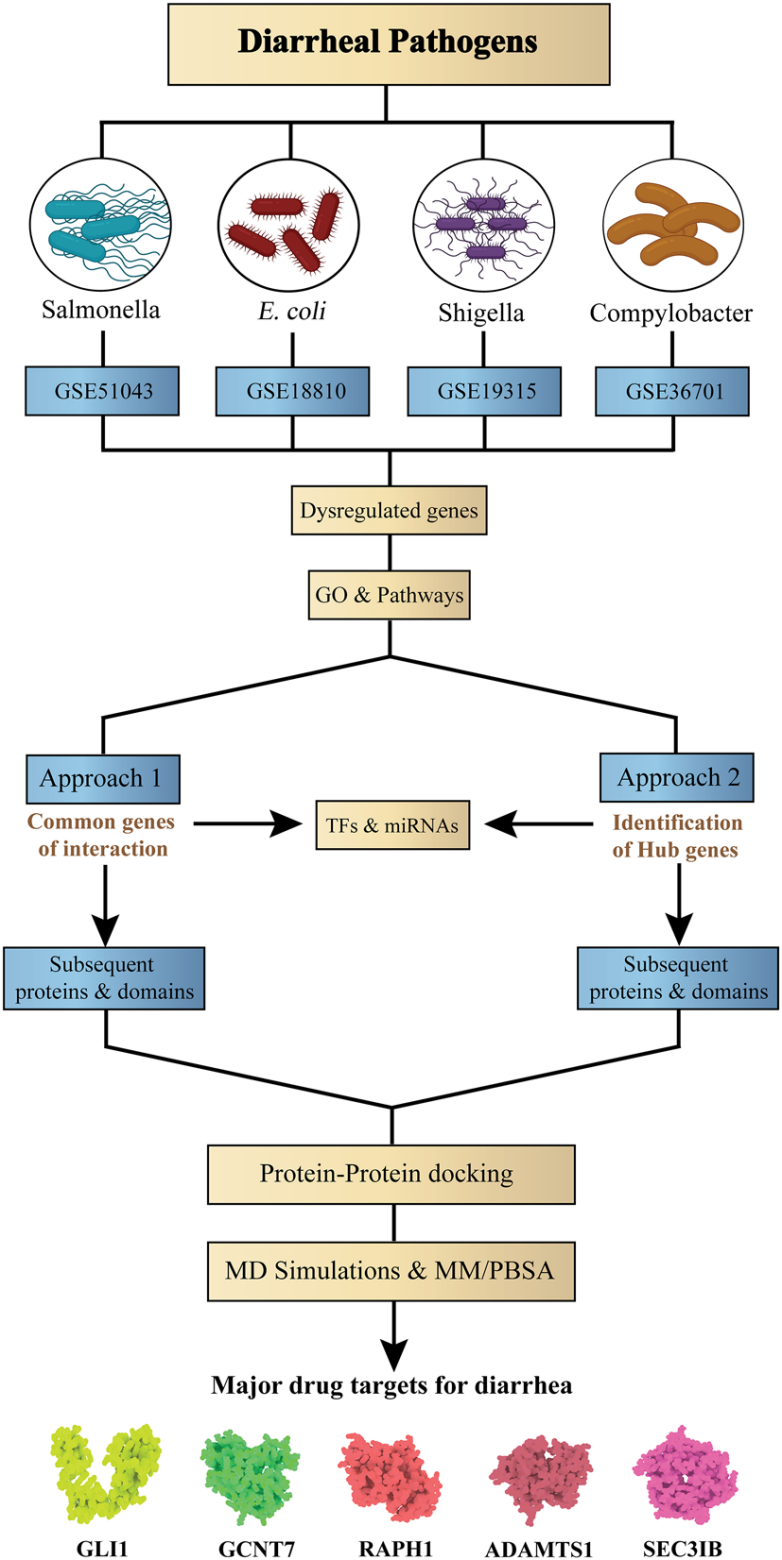
Schematic workflow of identifying the major druggable targets of diarrheal pathogens.

**Figure 2:**
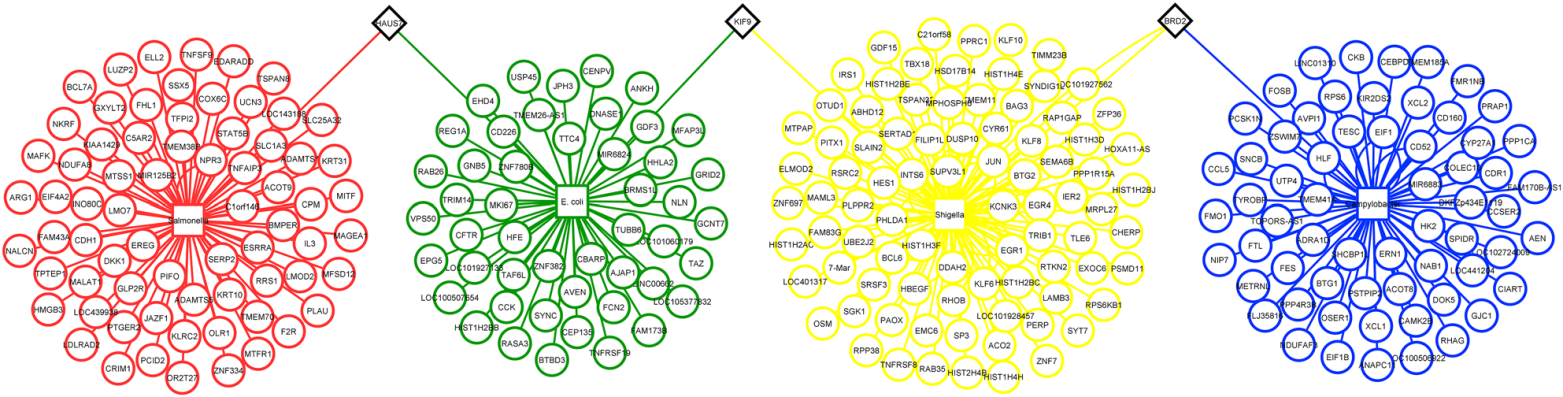
The common upregulated DEGs (black colored diamond boxes) found in the pathogenesis of four bacterial species. DEGs from *Salmonella, E. coli, Shigella,* and *Campylobacter* infection are shown in red, green, yellow, and blue colors, respectively.

**Figure 3:**
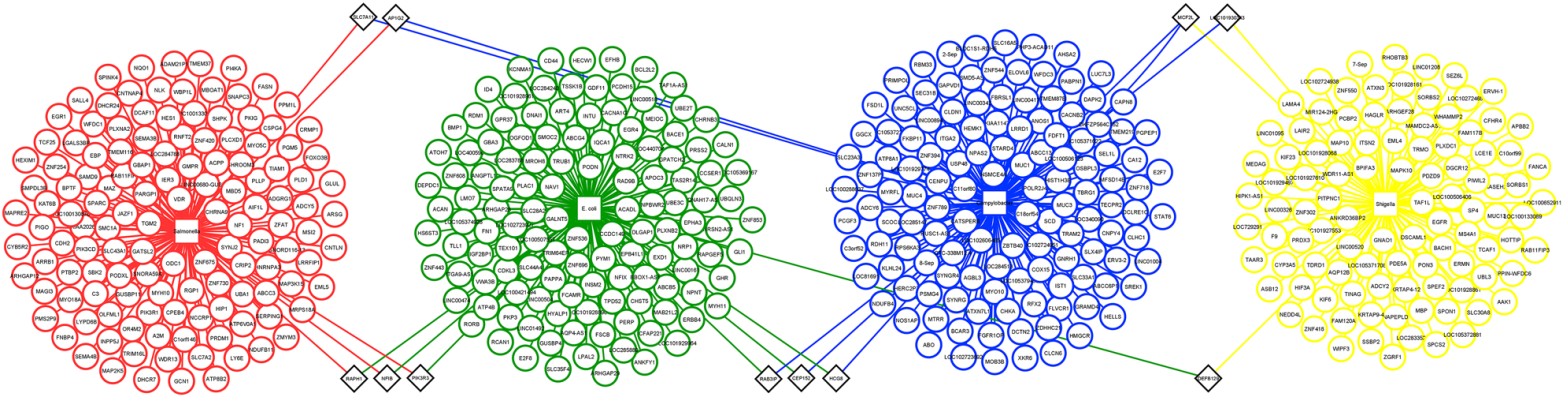
The common downregulated DEGs (black colored diamond boxes) found in the pathogenesis of four bacterial species. DEGs from *Salmonella, E. coli, Shigella,* and *Campylobacter* infection are shown in red, green, yellow, and blue colors, respectively.

The Cytoscape software v3.8 and the InteractiVenn tool (http://www.interactivenn.net/) were used to identify the common up and downregulated genes from the pathogenesis of four bacterial species. There was no single common DEG found in all four categories. The highest number (3) of commonly downregulated genes were found from *S.* Typhimurium-*E. coli* and *E. coli*-*C. jejuni* infections. The result is depicted in Figs. 1, 2, 3.

### Protein–protein (PPI) networks and identified hub proteins

Two PPI networks have been built from all the up-and downregulated gene interactions, shown in Supplementary file 6 (supplementary Fig. 1–2). Genes that often interact with other genes are known as hub genes in the gene networks. Hub genes typically play an essential function in a biological system due to these interactions. The protein–protein interaction (PPI) network, which is composed of highly connected (hub) genes, has a biological role demonstrated by the centrality-lethality rule^19^. Further, we employed four methods to determine the hub proteins within each group of proteins that differentially regulate pathogenesis by four bacteria. Each method identified the top 10 hub nodes within the PPI network. Except for the upregulated proteins resulting from *Campylobacter* and *Salmonella* pathogenesis, more than one hub protein was found to be common in all four methods within each group. The list of these hub proteins is tabulated in Table 2.

**Table 2:** The name and selection methods of the potential hub-proteins.

Causative pathogen	Regulation mechanism	Selection methods	Hub proteins	Name
Salmonella	Upregulation	MCC, MNC, degree	OLR1	Oxidized low-density lipoprotein receptor 1
			JAZF1	Juxtaposed with another zinc finger protein 1
			ADAMTS1	A disintegrin and metalloproteinase with thrombospondin motifs 1
	Downregulation	MCC, MNC, DMNC, degree	PIK3R1	Phosphatidylinositol 3-kinase regulatory subunit alpha
			TIAM1	Rho guanine nucleotide exchange factor TIAM1
*E. coli*	Upregulation	MCC, MNC, DMNC, degree	GCNT7	Beta-1,3-galactosyl-O-glycosyl-glycoprotein beta-1,6-N-acetylglucosaminyltransferase 7
			GNB5	Guanine nucleotide-binding protein subunit beta-5
	Downregulation	MCC, MNC, DMNC, degree	CD44	CD44 antigen
			GLI1	Zinc finger protein GLI1
			TAS2R14	Taste receptor type 2-member 14
Shigella	Upregulation	MCC, MNC, DMNC, degree	HIST1H2BC	Histone H2B type 1-C/E/F/G/I
	Downregulation	MCC, MNC, DMNC, degree	ADCY2	Adenylate cyclase type 2
			AAK1	AP2-associated protein kinase 1
Campylobacter	Upregulation	MCC, MNC, DMNC	RPS6	40S ribosomal protein S6
			ADRA1D	Alpha-1D adrenergic receptor
	Downregulation	MCC, MNC, DMNC, degree	RAB3IP	Rab-3A-interacting protein
			SYNRG	Synergin gamma
			SEC31B	Protein transport protein Sec31B

### Identified transcriptional and post-transcriptional biomarkers

**Figure 4:**
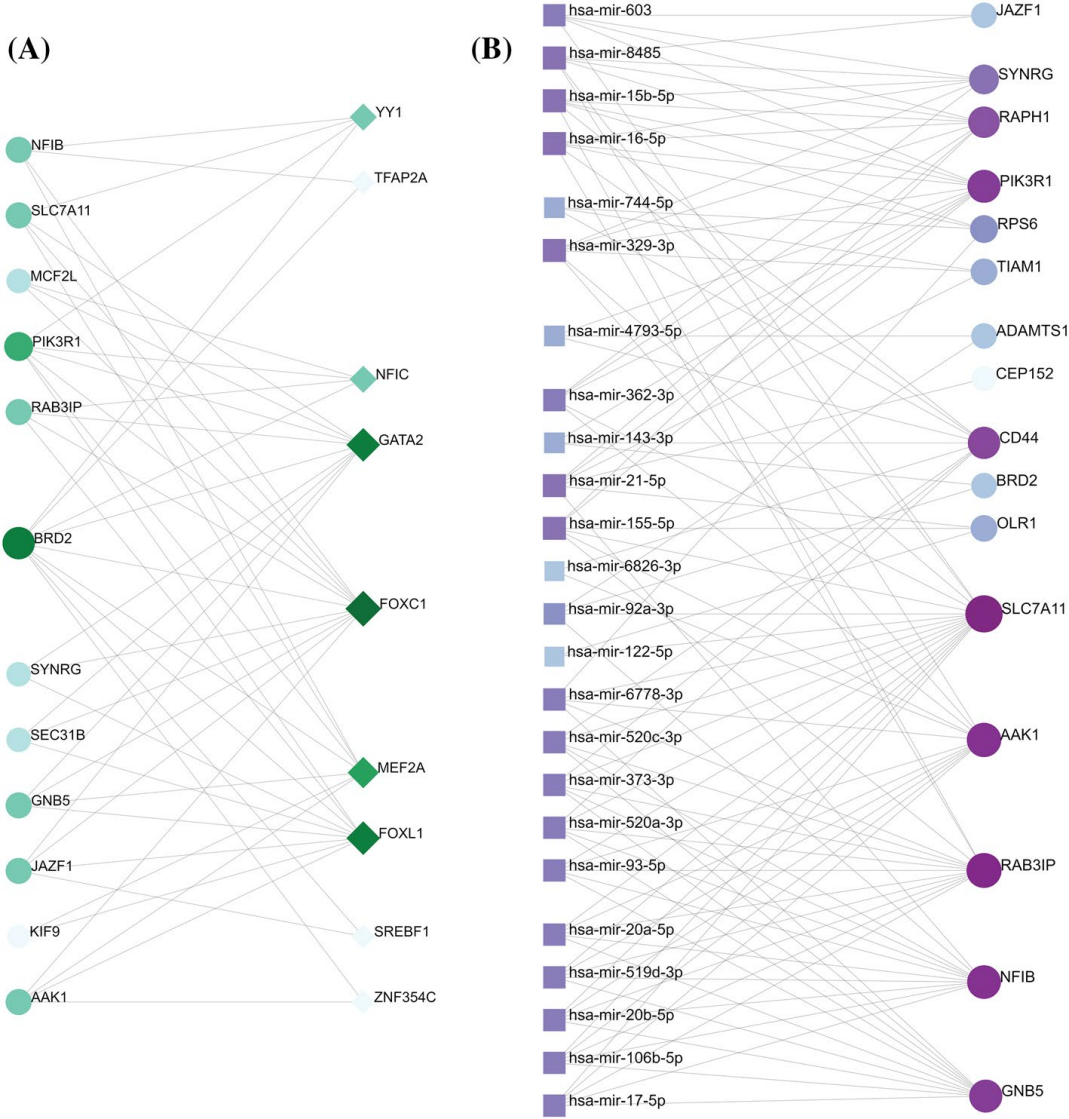
Gene regulatory networks associated with the dysregulated common and hub genes. The figure showing (**A**) gene–TF interacting network and (**B**) gene–miRNA interacting network. The interacting network of miRNAs and TFs were filtered with betweenness centrality ≥ 100 and 45, respectively.

Using the common and hub DEGs, we found 276 TFs (Transcriptional Factors) and 959 miRNAs (micro RNAs) that might influence the expression pattern of those genes and lead to the progression of diseases, as depicted in Fig. 4 and Supplementary File 8–9. Out of 276 TFs, the top TFs (i.e., ZNF354C, FOXC1, GATA2, FOXL1, YY1, MEF2A, NFIC, TFAP2A, SREBF1) were identified with betweenness centrality ≥ 45 as shown in Fig. 4A. Among all the miRNAs, we identified seventeen miRNAs (i.e., hsa-mir-17-5p, hsa-mir-20a-5p, hsa-mir-92a-3p, hsa-mir-93-5p, hsa-mir-122-5p, hsa-mir-155-5p, hsa-mir-106b-5p, hsa-mir-373-3p, hsa-mir-20b-5p, hsa-mir-329-3p, hsa-mir-520a-3p, hsa-mir-520c-3p, hsa-mir-519d-3p, hsa-mir-603, hsa-mir-362-3p, hsa-mir-6778-3p, hsa-mir-8485) with betweenness centrality ≥ 100 (Fig. 4B).

### Domains within the common and hub proteins and 3D structural modeling

NCBI’s (National Center for Biotechnology Information) conserved domain search tool revealed that several common and hub proteins were found to have multiple domains. In these cases, we selected one domain from each protein based on having a lower E-value, and the proteins that did not contain any functional domains predicted by this tool were excluded from this study. Out of the 26 domains, 3D structures of 8 domains were available in the RCSB PDB (Protein Data Bank) database, 12 of them were modeled through MODELLER 9.22, and 5 structures were modeled using the trROSETTA server due to having less than 40% query coverages. Table 3 contains the domain names, sequence length, e-values, and the method of 3D structure modeling of each domain. Later, all the modeled structures were refined using the GalaxyRefine server. The summary of quality assessment results (Ramachandran plot analysis, ERRAT server, and ProSA-Web analysis) of the refined structure are shown in Supplementary file 6 (Supplementary table 1), while Supplementary file 7 contains the Ramachandran plots of all the modeled structures.

**Table 3:** Predicted protein domains, sequence length, e-values and details of the structural modeling tools used to model their 3D structures.

Proteins	Domains	Sequence length	E-value	Structural modeling tools
Upregulated common proteins				
KIF9	Kinesin motor domain	6–338	0e + 00	Crystal structure (PDB ID: 3NWN)
BRD2	First bromodomain in Brdt_like superfamily	74–180	7.39e−79	MODELLER 9.22 (Template PDB ID: 6TQ1)
**Downregulated common proteins**				
NFIB	CTF/NFI DNA-binding domain	2–195	1.27e−19	(trROSETTA) De novo folding, guided by deep learning restraints
RAPH1	Ras-associating (RA) & pleckstrin homology (PH) domain	269–355	3.90e−48	Crystal structure (PDB ID: 4GN1)
PIK3R3	N-terminal Src homology 2 (nSH2) domain of p85	163–330	7.92e−91	MODELLER 9.22 (Template PDB ID: 6G6W_B)
AP1G2	Adaptin N terminal domain of AP-1	24–574	1.93e−178	MODELLER 9.22 (Template PDB ID: 6CRIG_G)
RAB3IP	Sec2p domain	191–261	4.62e−05	Crystal structure (PDB ID: 6F6P)
MCF2L	Dbs PH domain	816–947	1.10e−81	MODELLER 9.22 (Template PDB ID: 1LB1_A)
**Upregulated hub proteins**				
OLR1	C-type lectin-like domain	144–266	6.04e−41	MODELLER 9.22 (Template PDB ID: 6TLA_A)
JAZF1	PTRRG2/MT domain	169–230	8.50e−16	trROSETTA (De novo folding, guided by deep learning restraints)
ADAMTS1	Zinc-dependent metalloprotease	258–463	1.24e−112	MODELLER 9.22 (Template PDB ID: 2JIH_A)
GNB5	WD40 domain	56–99	4.20e−10	MODELLER 9.22 (Template PDB ID: 6N9G_C)
GCNT7	Core-2/I-Branching enzyme	111–374	3.06e−46	trROSETTA (Template PDB IDs: 2GAM_C, 2GAK_B, 3OTK_A, 6EJ7_A, 6FOA_A)
HIST1H2BC	Histone H2B	28–124	3.56e−53	Crystal structure (PDB ID: 6M4D)
RPS6	Ribosomal protein S6e	1–215	1.27e−102	MODELLER 9.22 (Template PDB ID: 3J7P_S)
ADRA1D	Alpha-1 adrenergic receptors subtype D domain	97–413	3.71e−102	trROSETTA (Template PDB IDs: 3VW7_A, 5T04_A, 5TGZ_A, 5X33_A, 3ODU_A)
**Downregulated hub proteins**				
PIK3R1	RhoGAP domain	114–302	2.26e−122	MODELLER 9.22 (Template PDB ID: 1PBW_A)
TIAM1	Pleckstrin Homology (PH) domain	1235–1406	6.08e−107	MODELLER 9.22 (Template PDB ID: 1FOE_A)
CD44	Hyaluronan (HA)-binding domain	26–170	3.88e−72	Crystal structure (PDB ID: 4PZ4)
GLI1	FOXP coiled-coil domain	237–265	3.27e−06	Crystal structure (PDB ID: 2GLI)
TAS2R14	TAS2R subtype 14 domain	8–295	5.74e−150	trROSETTA (Template PDB IDs: 3VW7_A, 5T04_A, 5X33_A, 5ZKP_A, 4GRV_A)
ADCY2	Adenylate and Guanylate cyclase catalytic domain	878–1077	3.09e−80	MODELLER 9.22 (Template PDB ID: 1AZS_B)
AAK1	Catalytic domain of Numb-Associated kinase (NAK)-like Serine/Threonine kinases	42- 316	0e + 00	MODELLER 9.22 (Template PDB ID: 5L4Q_A)
RAB3IP	Rab11 binding domain	60–252	7.84e−150	MODELLER 9.22 (Template PDB ID: 4UJ3_E)
SYNRG	Eps15 homology domain	316–367	1.76e−13	Crystal structure (PDB ID: 2MX7)
SEC31B	WD40 domain	13–332	2.21e−28	trROSETTA (Template PDB IDs: 4BZK_C, 6BM0_B, 5NZV_C, 3JCT, 6T9K_D)

### Binding interactions of dysregulated protein domains

**Figure 5:**
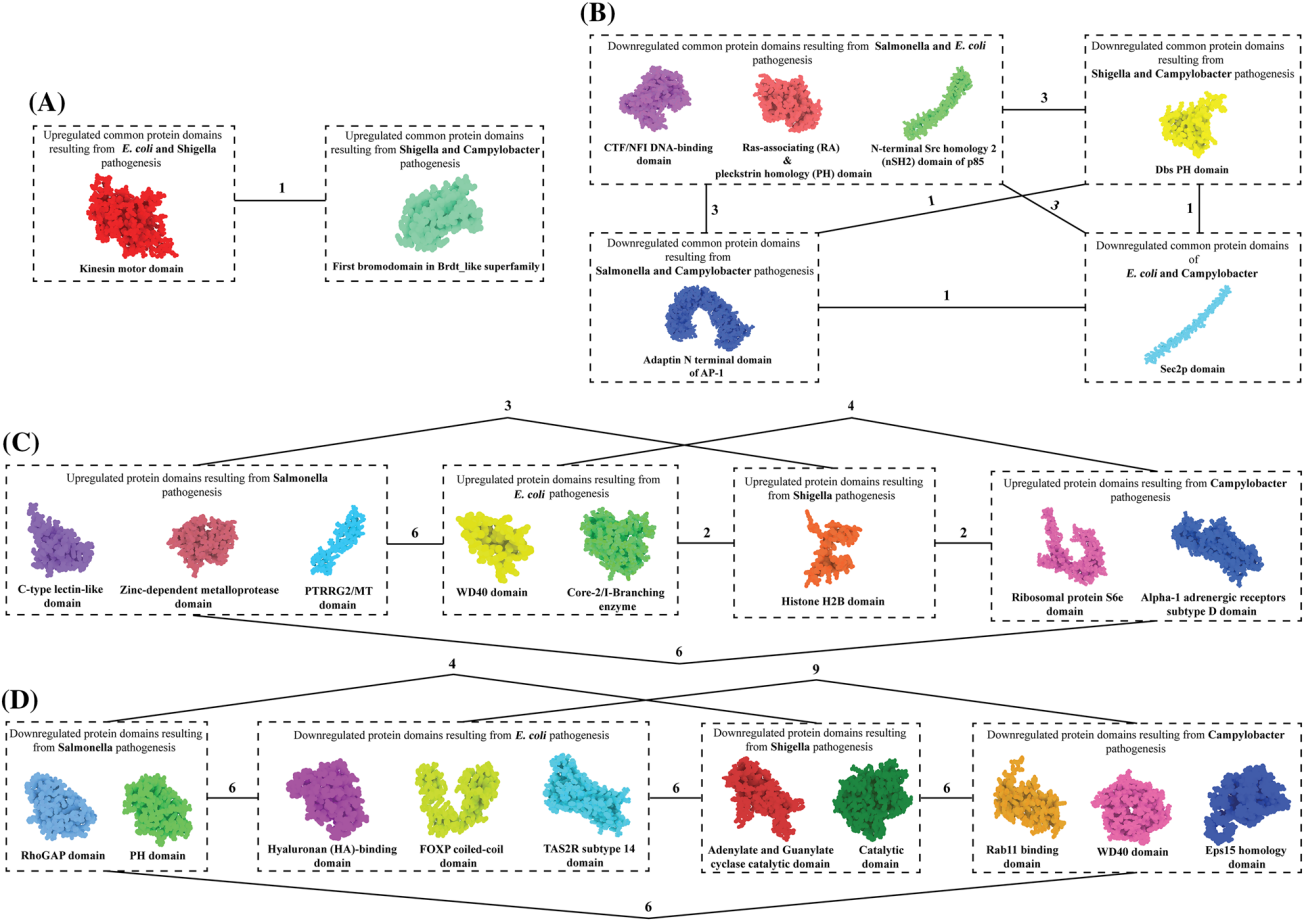
This study's schematic representation of domain-specific molecular docking protocol. The figure shows docking between (**A**) common upregulated protein domains, (**B**) common downregulated protein domains, (**C**) domains of upregulated hub proteins, and (**D**) domains of downregulated hub proteins. The black line and the numbers represent molecular docking and the numbers of possible docking combinations among them, respectively. From this combination, a total number of 73 molecular dockings were performed to elucidate their binding affinity.

The domain-specific protein–protein docking was performed to anticipate their binding affinity and interactions. The docking protocol is shown in Fig. 5 as a schematic representation. In doing so, the ClusPro v2.0 server provided up to 30 docked complexes with different poses. The complex with the least energy score and binding pose with functional interactions were selected from each docking process. It was found that the alpha-1 adrenergic receptors subtype D domain-zinc dependent metalloprotease domain complex showed the highest docking energy score of –1300 kcal/mol. The highest number (29) of hydrogen bonds were present in FOXP coiled-coil domain-PH domain complex. In contrast, the highest number (9) of salt-bridges were present in the interacting plane of core-2/I-branching enzyme-ribosomal protein s6e domain complex. The docking energies, several formed hydrogen bonds, and salt bridges of the complexes are enlisted in Supplementary file 6 (supplementary table 2).

### Stability of the docked complexes and potential drug targets identified for a single therapy

**Figure 6:**
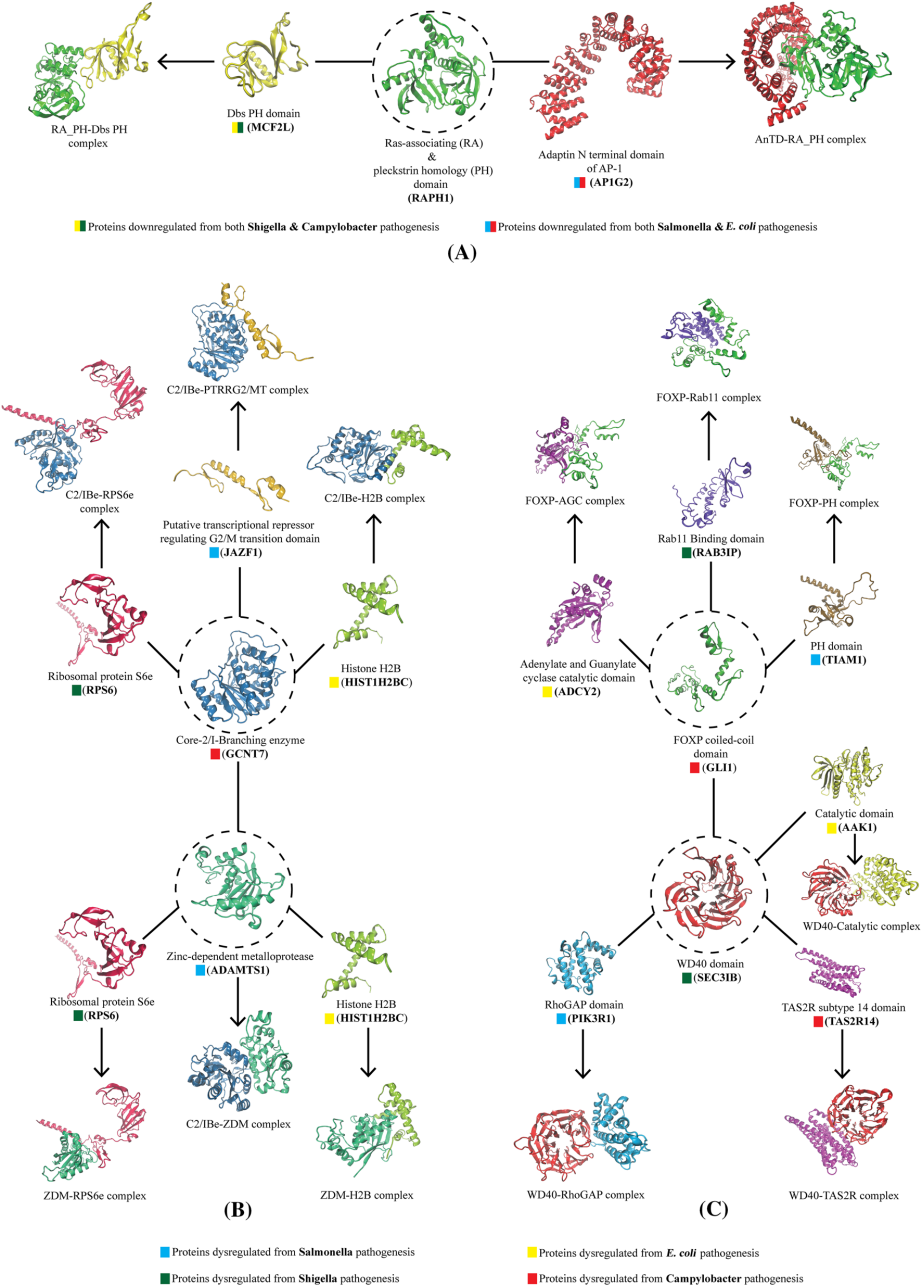
The 5 potential drug targets (shown in dotted circle) for single therapy. These 5 proteins have stable interaction with other proteins resulting from dysregulation by the pathogenesis of any of the four bacteria. The figure showing (**A**) the first drug target, RAPH1, which has stable interaction with MCF2L and AP1G2 proteins that downregulates from *Shigella*-*Campylobacter* and *Salmonella*-*Campylobacter* pathogenesis, respectively. (**B**) The second group of drug targets, GCNT7, and ADAMTS1, which upregulates from *E. coli* and *Salmonella* infections, had stable interaction with HIST1H2BC and RPS6 proteins that upregulates from *Shigella* and *Campylobacter* infection. GCNT7 was also found to be stable with ADAMTS1 and JAZF1, which are the upregulated hub proteins resulting from *Salmonella* pathogenesis. (**C**) The third group of drug targets, GLI1 and SEC31B, downregulates from *E. coli* and *Campylobacter* infection. GLI1 showed a stable association with ADCY2, RAB3IP, TIAM1, and SEC31B that downregulates from *Shigella*, *Salmonella,* and *Campylobacter,* respectively, while SEC31B had stability with PIK3R1, AAK1, and TAS2R14 downregulating from *Salmonella*, *Shigella,* and *E. coli* infection.

We employed molecular dynamics simulation to verify the stability of the docked protein complexes and to identify the common drug targets having a high association with significant proteins dysregulated from the pathogenesis of all four bacteria. A 7.3 microseconds (µs) production run was performed to simulate the 73 complexes. Out of them, 20 complexes remained stable while the others either got unstable several times during the simulation period or remained completely disassociated at the end of the simulation. From these analyses, we identified 5 proteins (Fig. 6) that might be targeted for single therapy as each has an association with significant proteins dysregulated from the pathogenesis of other bacteria. The first identified protein is RAPH1, a common protein that downregulates from *S. Typhimurium* and *E. coli* infections. RAPH1 showed the stable interactions with MCF2L and AP1G2 proteins, which was downregulated from *S. dysenteriae-C. jejuni* and *S*. Typhimurium-*C. jejuni* infections, respectively (Fig. 6A). The second group of drug targets identified was GCNT7 and ADAMTS1, upregulated from *E. coli* and *S.* Typhimurium infection, respectively. Both had stable interaction with HIST1H2BC and RPS6 proteins which were upregulated from *S. dysenteriae* and *C. jejuni* infection (Fig. 6B). Further stable association of GCNT7 was found with ADAMTS1 and JAZF1 also, which are the upregulated hub proteins resulting from *S.* Typhimurium infection. The following 2 plausible drug targets found were GLI1 and SEC31B, downregulated in the human body from *E. coli* and *C. jejuni* infection, respectively (Fig. 6C). GLI1 had a stable association with ADCY2, RAB3IP, TIAM1, and SEC31B that downregulates from *S. dysenteriae*, *S*. Typhimurium and *C. jejuni*, respectively, while SEC31B had stability with PIK3R1, AAK1, and TAS2R14 which was downregulated from *S. dysenteriae*, *S*. Typhimurium and *E. coli* pathogenesis (Fig. 6C). Thus, targeting these 5 drug targets with a single therapy might be a remarkable solution to prevent diarrheal disease from any four bacteria species.

**Figure 7:**
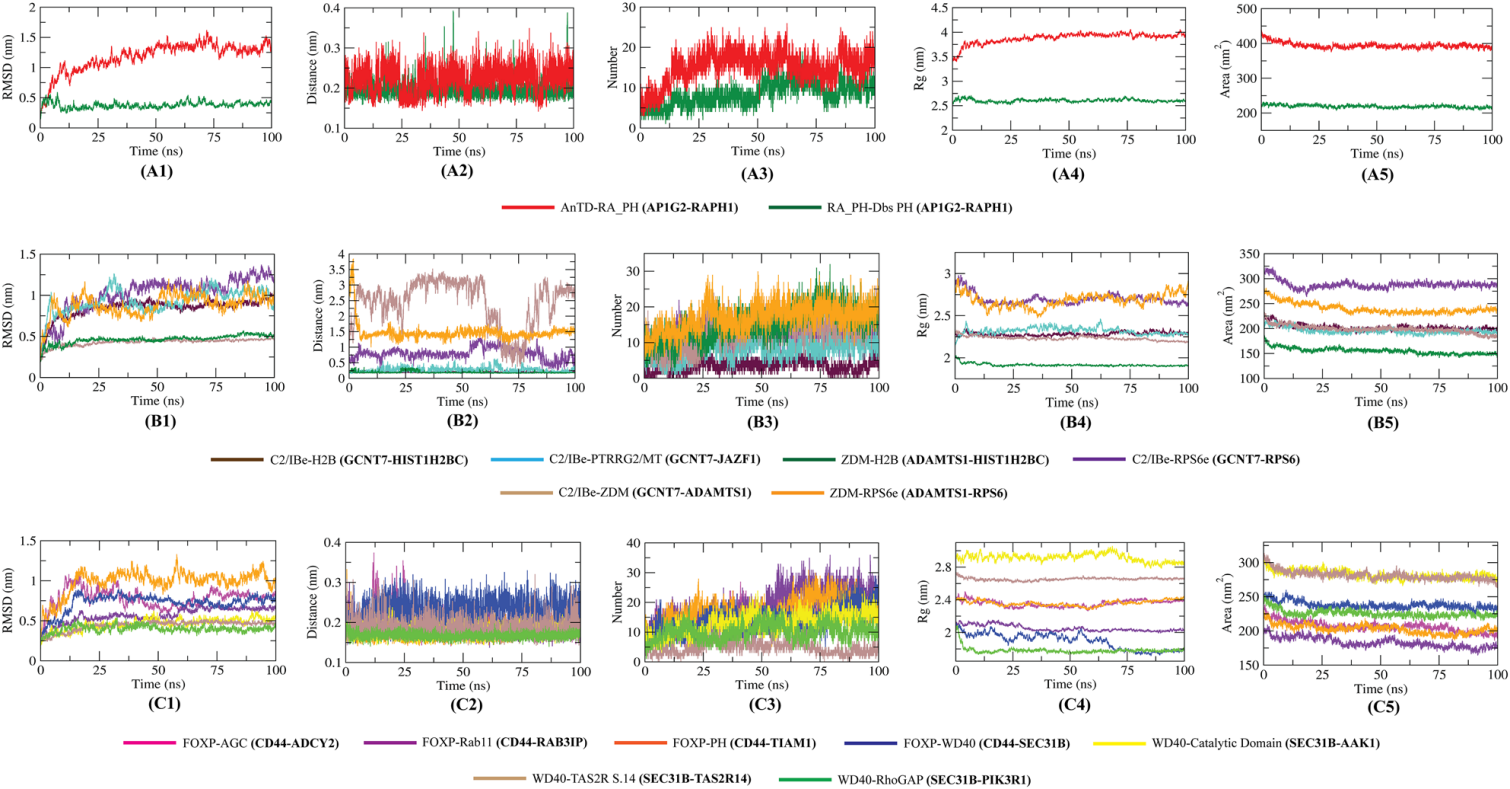
MD simulation results of protein complexes. The figure shows RMSD analysis (**A1**, **B1**, **C1**), minimum distances in the interacting residues of the complexes (**A2**, **B2**, **C2**), number of hydrogen bonds (**A3**, **B3**, **C3**), Rg analysis (**A4**, **B4**, **C4**), and SASA analysis (**A5**, **B5**, **C5**).

The dynamic behavior of the protein complexes was analyzed by RMSD, atomic distances of the interacting planes, number of hydrogen bonds, Rg, and SASA analysis (Fig. 7).

All protein complex configuration changes were analyzed in terms of RMSD during the simulation periods. Fig. 7(A1, B1, C1) shows that rather than some fluctuations in the AnTD-RA_PH, C2/Ibe-RPS6e, and FOXP-PH complexes, the RMSD values of the remaining complexes were quite stable. Although some fluctuations were observed in the C2/Ibe and FOXP bound complexes, they tend to stabilize after 75 ns (Figs. 7B1 and 7C1). We measured the changes in the minimum distances between the residues of interacting planes of the complexes during the simulation (Fig. 7A2, 7B2, 7C2). The high distance was observed in the RPS6e bound C2/IBe and ZDM complexes as well as in the C2/IBe-ZDM complex (Fig. 7B2). Rather than these, all the complexes showed a significantly less distance (< 0.3 nm) between the interacting residues throughout the simulation. We also calculated the number of hydrogen bonding interactions formed during the simulations between the domains of the protein complexes, as shown in Fig. 7 (A3, B3, C3). Hydrogen bonding is one of the primary components responsible for molecular interactions in any biological system. In the FOXP-Rab11 complex, the highest number of conformations formed up to 30 hydrogen bonds during the simulation (Fig. 7C3). Very few conformations formed less than five hydrogen bonds in the rest of the complexes. Further, we measured the radius of gyration (Rg) for the protein complexes contributing to their compactness shown in Fig. 7(A4, B4, C4)**.** It can be inferred that all the complexes had approximately similar compactness as to their starting structure except the AnTD-RA_PH complex, where a higher Rg score was observed (Fig. 7A4). Finally, the solvent-accessible surface areas (SASAs) were analyzed to investigate the changes in the protein volumes upon association of complexes (Fig. 7A5, 7B5, 7C5). Interesting results were observed as almost all the complexes showed slightly decreased SASA values compared to the starting point of the simulation. These decreased SASA values in the protein complexes denote a relatively shrunken nature upon their association. Supplementary movies 1–15 visualizes the 100 ns MD simulation of these 15 complexes related to 5 identified drug targets (the color codes of proteins are the same as shown in Fig. 6). The simulation analysis of the remaining 5 stable complexes and the RMSD analysis of the 53 unstable complexes are shown in supplementary file 6 (supplementary Fig. 4).

### Binding energies among the protein complexes

We calculated the binding energy of the last 20 ns of MD production run of the protein complexes (associated with 5 identified drug targets) with an interval of 50 ps from MD trajectories using the MM/PBSA method. Further, we utilized the MmPbSaStat.py script for calculating the average free binding energy and its standard deviation/error from the output files obtained from the g_mmpbsa package (Table 4). The interaction between the two proteins is shown in the form of binding energy, where the lesser the binding energy, the better the binding of the two proteins. The final binding energy is the result of the cumulative sum of van der Wall, electrostatic, polar solvation, and SASA energy. The majority of the complexes showed favorable binding energy between them, which further validates their stability, and the ZDM-RPS6E complex (ADAMTS1-RPS6) showed the least binding free energy (-3060.937 kJ/mol) among all the complexes.

**Table 4:** MM/PBSA calculations of binding energy for the 14 protein complexes associated with the 5 significant drug targets.

Gene–gene interactions	Corresponding protein domain-domain complexes	Van der Waal energy (KJ mol^−1^)	Electrostatic energy (KJ mol^−1^)	Polar solvation energy (KJ mol^−1^)	SASA energy (KJ mol^−1^)	Binding energy (KJ mol^−1^)
**Downregulated common proteins (domains)**						
RAPH1-AP1G2	RA_PH-AnTD	− 680.087 ± 37.247	− 439.522 ± 64.004	1474.158 ± 194.497	− 86.160 ± 8.274	268.390 + / 165.384
RAPH1-PIKR3	RA_PH-Dbs PH	− 189.003 ± 65.551	− 452.402 ± 231.165	839.545 ± 333.576	− 24.579 ± 9.925	173.560 + / 123.451
**Upregulated hub proteins (domains)**						
GCNT7-RPS6	C2/IBe-RPS6e	− 493.664 ± 37.128	2758.720 ± 108.689	1263.838 ± 158.046	− 62.830 ± 6.490	466.065 ± 148.483
GCNT7-HIST1H2BC	C2/IBe-H2B	− 227.021 ± 113.840	421.572 ± 51.336	437.592 ± 205.328	− 27.198 ± 13.569	604.945 ± 113.255
GCNT7-JAZF1	C2/IBe-PTRRG2/MT	− 139.229 ± 103.401	247.097 ± 37.191	334.411 ± 230.916	− 20.305 ± 16.120	421.974 ± 151.540
GCNT7-ADAMTS1	C2/IBe-ZDM	− 404.509 ± 408.908	− 1191.466 ± 422.802	814.694 ± 742.721	− 36.994 ± 37.970	− 818.276 ± 160.821
ADAMTS1-RPS6	ZDM-RPS6E	− 658.815 ± 35.537	− 4263.268 ± 163.136	1944.392 ± 159.166	− 83.247 ± 5.610	− 3060.937 ± 158.136
ADAMTS1-HIST1H2BC	ZDM-H2B	− 547.580 ± 218.170	− 1480.476 ± 444.787	1673.507 ± 673.833	− 76.334 ± 30.324	− 430.883 ± 94.673
**Downregulated hub proteins (domains)**						
GLI1-ADCY2	FOXP-AGC	− 342.016 ± 296.396	− 962.872 ± 245.660	797.151 ± 488.452	− 45.236 ± 37.246	− 552.973 ± 164.306
GLI1-TIAM1	FOXP-PH	− 70.478 ± 175.026	− 156.009 ± 244.743	277.115 ± 587.029	− 9.406 ± 24.011	41.222 ± 169.806
GLI1-RAB3IP	FOXP-Rab11	− 642.355 ± 198.352	− 921.736 ± 296.892	1618.073 ± 539.597	− 82.793 ± 25.178	− 28.811 ± 154.599
GLI1-SEC31B	FOXP-WD40	− 638.625 ± 34.584	− 966.445 ± 73.608	1578.224 ± 173.598	− 79.436 ± 6.530	− 106.282 ± 128.871
SEC31B-TAS2R14	WD40-TAS2R subtype 14	− 283.437 ± 217.294	− 614.752 ± 378.329	252.006 ± 241.116	− 29.140 ± 25.235	− 675.323 ± 403.508
SEC31B-PIK3R3	WD40-RhoGap	− 46.216 ± 50.728	56.612 ± 22.919	79.744 ± 87.659	− 6.394 ± 7.951	83.747 ± 39.309
SEC31B-AAK1	WD40-Catalytic	− 322.391 ± 33.217	− 272.767 ± 42.810	855.395 ± 97.526	− 41.380 ± 6.579	218.856 ± 88.269

## Discussion

Examining the molecular mechanisms that drive illness onset and progression is generally focused on biomolecules such as genes and proteins, whose abnormal expression contributes to changes in cellular function and, eventually, disease. By concentrating on illness molecular pathways, researchers can uncover crucial events that can be addressed with novel therapy techniques. Identifying these disease-associated targets is thus an important first step in disease mechanism research.

We focused on the causal agents of diarrheal disease in this study, specifically *S*. Typhimurium, *C. jejuni*, *E. coli*, *S. dysenteriae* (Fig. 1). After being infected by these bacteria, a large number of genes in the human body become dysregulated, either individually or as a group. From four datasets available through the Gene Expression Omnibus database, we found 298 upregulated and 529 downregulated genes (Supplementary files 1–2). The pathways that may play a critical role in illness development were chosen. Notably, the majority of these genes and pathways have been linked to cancer (Supplementary File 3–4). Cancer pathways, cytokine-cytokine receptor interactions, chemical carcinogenesis, Rap1 signaling pathway, and nuclear receptors meta-pathway are among the predicted pathways (Table 1 and supplementary file 5). Although diarrhea is a common and often dose-limiting complication associated with cancer chemotherapy treatment, it is underappreciated and poorly handled^20^. CS diarrhea affects 80 percent of Carcinoid syndrome (CS) patients, who experience diarrhea and flushing, necessitating considerable modifications in daily activities and lifestyle^21^. Proinflammatory cytokines are known to increase in diarrhea-predominant irritable bowel syndrome patients, which could explain why cytokine-cytokine receptor interactions are occurring in expected pathways^22^. Ras-associated protein 1 (Rap1) is triggered by various stimuli in the Rap1 signaling pathway. It then recruits several effectors, resulting in its involvement in essential physiological processes such as integrin signalling and ERK activation^23^. Epac, a family of intracellular cAMP sensors, activates Rap1 by accelerating the conversion of GDP-Rap1 to GTP-Rap1. In contrast, active GTP-Rap1 may have a role in the pathophysiology of secretory diarrhea via the RhoA-Rho-associated kinase (ROCK) pathway^24^. Like pathways, GO terms pathways include the cell population proliferation, RNA polymerase II cis-regulatory region sequence-specific DNA binding, nervous system development, and cis-regulatory region sequence-specific DNA binding, which are relevant to diarrhea.

Based on the comparative analysis, we identified the common genes among the upregulated and downregulated DEGs (Fig. 2, 3). It was found that the highest number of three genes were common among the downregulated DEGs resulting from *S.* Typhimurium*-E. coli* and *E. coli-C. jejuni* infections (Fig. 2). Two PPI networks were built using the DEGs to display their relationship and identify the key disease modulators in diarrhea (supplementary Fig. 1–2). The centrality-lethality rule states that deleting a protein node that is highly connected (a "hub") is more likely to be fatal to an organism than deleting a node that is weakly connected (a "non-hub"). The centrality-lethality rule is commonly regarded to reflect the role of network design in defining network function since hubs are more important than non-hubs in organizing the global network structure. Hub proteins have eight or more interactions, whereas non-hub proteins have four or fewer interactions^25^. Because they have many interacting partners within a network, hub proteins are considered functionally significant^26^. We identified eighteen hub proteins (OLR1, JAZF1, ADAMTS1, PIK3R1, TIAM1, GCNT7, GNB5, CD44, GLI1, TAS2R14, HIST1H2BC, ADCY2, AAK1, RPS6, ADRA1D, RAB3IP, SYNRG, SEC31B) implicated in diarrheal pathogenesis by each of the four bacteria using various approaches (Table 2). TFs also influence the rate of transcription^27^, and miRNA is involved in RNA silencing and gene expression regulation at the post-transcriptional level^28^. As a result, both are necessary to comprehend the progression of a certain disease. We identified several TFs, such as NFIC, FOXC1, FOXL1, and ZNF345, which are known to be involved in DNA-binding transcription factor activity^29–32^ (Fig. 4 and supplementary file 8). The remaining transcription factors, such as YY2, TFAP2A, GATA2, MEF2A, and SREBF1, are implicated in positive and negative regulation of the transcription of several target genes, branchiooculofacial syndrome (BOFS), development and proliferation of hematopoietic and endocrine cell lineages, muscle development, neuronal differentiation, cell growth control, and apoptosis as well as sterol biosynthesis^33–36^. Moreover, YY1, GATA2, MEF2A, FOXC1, and SREBF1 were found to involve in Irritable Bowel Syndrome (IBS)^37–41^. Mir-92a-3p, mir-122-5p, mir-143-3p, mir-106b-5p, and mir-6826-3p were found to be linked to lupus erythematosus^42^, lipoprotein metabolism^43^, acute ischemic stroke^44^, chronic thromboembolic pulmonary hypertension^45^, and neuronal function loss^46^ among the 24 miRNAs studied (supplementary file 9). Gastric, cervical, pancreas, lung, colon, colorectal, thyroid, ovarian, prostate, hepatocellular carcinoma, osteosarcoma, and testicular germ cell malignancies were all involved in the remaining 19 miRNAs^47–50^. Also, previous research showed that mir-15, miR-16, miR-125b, and mir-106b were involved in Irritable Bowel Syndrome (IBS) with diarrhea^51,52^ while miR-143, miR-145, miR-21, miR-155, miR-21, miR-92a, miR-122, miR-17, mir-106a and mir-362-3p were found to involved in ulcerative colitis and other gastrointestinal tract diseases^53–59^.

Identifying common proteins and hub proteins in each of the four protein groups dysregulated by four bacterial infections prompted us to investigate the possibility of a functional relationship between them to understand their co-regulation better. We identified the domains inside each protein and used multiple up-to-date modeling tools to model their 3D structures before subjecting them to molecular docking utilizing an integrated procedure (Figs. 5, 6, Table 3 and supplementary file 7). Among the protein complexes, we found a wide range of binding energies, hydrogen bonds, and salt bridges (supplementary table 1). So, in order to confirm the docking complexes' stability and get a better understanding of their possible interaction and co-regulation, we performed molecular dynamics simulations on all 73 protein complexes and observed that just 20 of them are stable in the solvated state. This explains that only 20 of these 73 protein complexes might co-regulate (either upregulation or downregulation) during diarrhea by four bacterial pathogenesis. Following these investigations, we identified five proteins (RAPH1, GCNT7, ADAMTS1, GLI1, and SEC31B) that have a stable, functional relationship with the other hub proteins resulting from the pathogenesis of four bacteria (Fig. 6).

The RMSD analysis revealed that all of the complexes were stable and showed reduced changes during the simulation period, based on the interpretation of post-MD data (Fig. 7A1, 7B1, 7C1). Except for the C2/Ibe-RPS6e and C2/IBe-ZDM complexes, measurements of changes in the minimum distances between the residues of interaction planes of the complexes during simulation revealed that the remaining complexes had a distance of less than 0.3 nm between the interacting residues (Fig. 7A2, 7B2, 7C2). The stability of the protein complexes was further validated by a sufficient number of H-bond estimations during MD simulation (Fig. 7A3, 7B3, 7C3). The gyration radius revealed that the protein complexes maintained a consistent level of compactness across time (Fig. 7A4, 7B4, 7C4). The SASA study revealed that the complexes obtained less volume as a result of their interaction, which could be the cause of protein functional alterations (Fig. 7A5, 7B5, 7C5). The MM/PBSA results revealed that the majority of the proteins bind efficiently among themselves, as evidenced by good binding free energies (Table 4). In particular, the ADAMTS1 protein exhibited the most efficient binding energies with the GCNT7, RPS6, and HIST1H2BC proteins, which are upregulated hub proteins resulting from pathogenesis by *E. coli*, *C. jejuni* and *S. dysenteriae* respectively, while the ADAMTS1 protein itself is upregulated by *S*. Typhimurium (Table 4). These analyses make ADAMTS1 the most plausible therapeutic target of all the five identified proteins for the development of common drugs against diarrhea.

The biological function and activity of a cell are driven by switching on and off gene expression. Conversely, gene transcription is a facilitator of the pathogenic events that drive the evolution and progression of the disease, as well as directing the response to therapy. By comparing gene expression profiles under different disease conditions, individual genes or their corresponding protein products can be identified as therapeutic targets. Our study reports five such proteins, RAPH1, GCNT7, ADAMTS1, GLI1, and SEC31B, which are strong binding partners of other significant proteins and could thus be targeted for the discovery of a common medication system against diarrheal disease.

## Material and methods

A step-wise protocol consisting of two approaches was followed to identify the major druggable targets against the four diarrheal pathogens. The workflow is depicted in Fig. 1.

### Retrieval of microarray data

Gene Expression Omnibus (GEO) is an internationally acclaimed online database^60^ (https://www.ncbi.nlm.nih.gov/geo/) by National Center for Biotechnology Information (NCBI) for high-throughput sequencing data, microarray, and hybridization array data. We downloaded four datasets from this database with accession numbers GSE51043 (GSM1236481-GSM1236489)^6^ for *S.* Typhimurium, GSE18810 (GSM466514-GSM466519)^61^ for*E. coli*, GSE19315 (GSM479983-GSM479991)^62^ for *S. dysenteriae*, and GSE36701 (GSM899034-GSM899254)^63^ for *C. jeju*ni for this study. Further, the limma^64^ and DESeq2^65^ package of R was used to analyze these datasets. We used the False Discovery Rate (FDR) to find the dysregulated genes from the RNA-seq data analysis. The amount of gene expression between the control and sample was determined using the statistical criterion of log2fold change (bacteria-infected patients). The log2fold change parameters (2 and − 2) were adjusted to reflect the higher significant upregulated and downregulated genes, which indicate that the genes are dysregulated (upregulated and downregulated). Following that, we also employed a statistically significant P (probability) value (P ≤ 0.05) to identify genes that were dysregulated because a P value larger than 0.05 indicates that no difference between the control and the sample was seen.

### Gene ontology (GO) and pathway enrichment analysis of differentially expressed genes (DEG)

The signaling pathways and gene ontologies associated with the up-and downregulated genes were predicted using different databases via the Enrichr enrichment analysis online tool^66^ (https://maayanlab.cloud/Enrichr/). For pathways, we considered KEGG^67,68^ (2021), while biological process (2021) and molecular function (2021) were evaluated for gene ontologies. The significant pathways were filtered using the p-value with a cutoff score set to 0.05.

### Identification of common gene expression signature in DEGs

We identified the common dysregulated (both upregulated and downregulated) genes from four bacteria using the Cytoscape software v3.8^69^ the InteractiVenn tool (http://www.interactivenn.net/). The name of the bacterial species was set as a node, and the DEGs were set as target nodes to generate the network. Further, the resulting network showed the common genes among the four species.

### Analysis of protein–protein interaction (PPI) and identification of hub protein networks

Protein–protein interaction (PPI) of the DEGs was analyzed using the STRING database^70^ with a confidence score of ≥ 0.4. The organism was specified as *H. sapiens*, and the generated PPI network was visualized using the Cytoscape software. We also generated a PPI network of all the up-and downregulated DEGs of four bacteria separately and determined potential hubs within these networks by applying different local-based methods using the cytoHubba^71^ plugin in Cytoscape v3.8. Based on the relationship between the node and its direct neighbor, the local method ranked the hub proteins. In total, four local rank methods were considered, i.e., maximal clique centrality (MCC), maximum neighborhood component (MNC), the density of maximum neighborhood component (DMNC), and degree method.

### Identification of regulatory biomolecules among the common and hub genes

Transcription factors (TFs) and microRNAs (miRNAs) are regulatory molecules responsible for significant changes in transcription and expression results. Therefore, we deployed experimentally verified JASPAR^72^ and miRTarbase v6.0^73^ datasets to anticipate TF–gene and miRNA–gene interactions via the NetworkAnalyst v3.0^74^ web tool. Both networks were visualized with Cytoscape v3.8.

### Identification and structural modeling of domains within the common and hub proteins

The distinct functional and structural units in a protein are domains responsible for a particular function contributing to the overall role of a protein^75^. The domains of analyzed significant hub proteins were predicted using NCBI’s CD-Search tool (https://www.ncbi.nlm.nih.gov/Structure/cdd/wrpsb.cgi))^76^. Further, the domains’ three-dimensional (3D) were searched and retrieved from the PDB database (https://www.rcsb.org/). The structures that were not present in the database were modeled using MODELLER 9.22^77^ and the trROSETTA^78^ sever based on query coverage and further refined through the GalaxyRefine^79^ server. The best structure from MODELLER was chosen using the DOPE and GA341 objective functions, where a higher GA341 and/or lower DOPE score indicates a high quality of a generated model. Further, the modeled structures were assessed using PROCHECK^80^ and ERRAT^80^ tools from SAVES 6.0 server and ProSA-web^81^ analysis program.

### Assessment of binding interactions between protein domains

Molecular docking analysis was performed to study the stable domain-domain interactions among all the proteins and related sub-cellular functions. The ClusPro 2.0: protein–protein docking server^82^ was used for this purpose. In this server, the PIPER docking program^83^ is used by the rigid body docking phase, which relies on the Fast Fourier Transform (FFT)^84^ correlation approach. PIPER depicts the interaction energy between two protein molecules using an expression of form E; E = w_1_E_rep_ + w_2_E_attr_ + w_3_E_elec_ + w_4_E_DARS,_ where, E_rep_ and E_attr_ represent the attractive and repulsive contributions to the van der Waals interaction energy, E elec is electrostatic energy, while E_DARS_^85^ is a pairwise structure-based potential that primarily represents desolvation contributions. The ClusPro 2.0 server can differentiate thousands of conformations of the protein on the basis of different desolvation and electrostatic potentials. Following the docking process, each complex with the least binding energy was submitted to the PDBsum^86^ server (http://www.ebi.ac.uk/thornton-srv/databases/pdbsum/Generate.html) to view the residues involved in the interacting planes.

### Molecular dynamics simulation and binding energy calculation

Molecular dynamics simulation of the docked protein complexes was performed using GROMACS 5.1.4^87,88^ version on Linux 5.4 package. The GROMOS96 54a7^89^ was the selected force field as this parameter set has the enhanced capacity of the backbone NH and CO groups to form hydrogen bonds with each other resulting in reproducing the folding equilibria slightly better and sampling more 314-helical or hairpin conformations than the previous 53A6 or 45A3 force fields^90^. The protein complexes were solvated using simple point charge (SPC) water molecules in a rectangular box where the required number of Na + and Cl − ions were added to electrically neutral the simulation system. Upon setting the salt concentrations to 0.15 mol/L, the solvated systems were subjected to energy minimization for 5000 steps using the steepest descent method. Afterward, an NVT (constant number of particles, volume, and temperature) ensemble and an NPT (constant number of particles, pressure, and temperature) ensemble were conducted at 300 K temperature and 1 atm for a duration of 100 picoseconds (ps) to equilibrate the systems. Throughout the simulation, V‐rescale and Parrinello‐Rahman were selected as the thermostat and barostat, respectively. Finally, the production runs of all the protein complexes were performed at 300 K for a duration of 100 ns (nanoseconds) in a GPU (Graphics processing unit) accelerated supercomputing system that was provided by the Bioinformatics Division of the National Institute of Biotechnology (NIB), Bangladesh. Thereafter, in order to evaluate the stability of the complexes, root mean square deviation (RMSD), root mean square fluctuation (RMSF), a number of hydrogen bonds, the radius of gyration (Rg), atomic distances, and solvent accessible surface area (SASA) were analyzed and represented in the form of plots using the Qtgrace program.

Further, to calculate the binding energies through the MM/PBSA (Molecular Mechanics/Poisson Boltzmann Surface Area) method, the g_mmpbsa^91^ package of GROMACS was used, followed by the final MD run to get a more detailed overview of the biomolecular interactions between the two domains in every protein complexes. The total ΔG_bind_ of each protein–protein complex was determined from the free solvation energy (polar and nonpolar solvation energies) and potential energy (electrostatic and Van der Waals interactions). The binding energies were calculated using the following equation in this method:

$$\Delta {\text{G}}_{{{\text{binding}}}} = {\text{G}}_{{{\text{complex}}}} - ({\text{G}}_{{{\text{protein1}}}} + {\text{G}}_{{{\text{protein2}}}} ).$$

Here, the ΔG_binding_ = the total binding energy of the protein–protein complex, G_protein1_ = the binding energy of the first protein, and G_protein2_ = the binding energy of the second protein.

### Ethics approval and consent to participate

Not applicable.

### Consent for publication

Not applicable.

## Conclusion

The identification of therapeutic targets is critical for the development of novel medications to treat pathogen-related disorders. According to our findings, five crucial genes from the CESS are likely candidates for common drug discovery against the CESS. The pharmaceutical and scientific communities may be interested in this innovative method through differential gene expression for identifying targets for future therapeutic development research.

## Supplementary Information

Supplementary Information 1.

Supplementary Information 2.

Supplementary Information 3.

Supplementary Information 4.

Supplementary Information 5.

Supplementary Information 6.

Supplementary Information 7.

Supplementary Information 8.

Supplementary Information 9.

Supplementary Information 10.

Supplementary Information 11.

## Data Availability

All data generated and analyzed during this study are included in this article.
