# Supplementary material for: Pathogen-driven gene expression patterns lead to a novel approach to the identification of common therapeutic targets
Source: Sci Rep. 2022 Dec 6;12:21070. doi: 10.1038/s41598-022-25102-8 (PMC9726901; doi:10.1038/s41598-022-25102-8)
Supplement: Supplementary file 1 — Supplementary Information 1. [file 41598_2022_25102_MOESM1_ESM.docx]

| **Modeled protein domains** | **Residues in most favoured region (%)** | **Residues in allowed region (%)** | **ERRAT quality score** | **Z-Score from ProSA-Web** |
| --- | --- | --- | --- | --- |
| First bromodomain in Brdt_like superfamily | 94.6 | 5.4 | 85.8586 | -6.29 |
| CTF/NFI DNA-binding  domain | 96.0 | 4.0 | 88.1081 | -7.63 |
| N-terminal Src homology 2 | 99.4 | 0.6 | 98.75 | -5.34 |
| Adaptin N terminal domain  of AP-1 | 97.6 | 2.0 | 94.4134 | -12.21 |
| Dbs PH domain | 94.8 | 4.3 | 90.0901 | -4.36 |
| C-type lectin-like domain | 91.3 | 7.8 | 97.3451 | -5.08 |
| PTRRG2/MT  domain | 100.0 | 0.0 | 100 | -2.65 |
| Zinc-dependent metalloprotease | 93.5 | 6.0 | 85.1282 | -6.98 |
| WD40 domain | 92.1 | 6.3 | 89.6552 | -2.42 |
| Core-2/I-Branching enzyme | 93.5 | 6.5 | 90.2439 | -7.27 |
| Ribosomal protein S6e | 94.5 | 4.8 | 65.098 | -4.81 |
| Alpha-1 adrenergic receptors subtype D domain | 94.2 | 5.8 | 94.3333 | -3.35 |
| RhoGAP domain | 96.4 | 1.2 | 97.1591 | -7.56 |
| Pleckstrin Homology (PH) domain | 91.1 | 7.3 | 68.125 | -6.36 |
| TAS2R subtype 14  domain | 100.0 | 0.0 | 100 | -3.38 |
| Adenylate and Guanylate cyclase catalytic domain | 97.7 | 2.2 | 82.4468 | -7.43 |
| Catalytic domain of Numb-Associated kinase (NAK)-like Serine/Threonine kinases | 93.7 | 6.3 | 96.0993 | -8.73 |
| Rab11 binding domain | 94.0 | 4.7 | 91.5033 | -5.85 |

**Supplementary table 1**: Ramachandran plot analysis, ERRAT and ProSA-Web analysis results of the modeled protein domains.

| **Protein domain-domain complex** | **Docking energy (Kcal/mol)** | **Number of hydrogen bonds** | **Number of salt bridges** | **Number of non-bonded interactions** |
| --- | --- | --- | --- | --- |
| ***Docking between Upregulated common protein domains*** | | | | |
| KMD-First bromodomain | -749.9 | 5 | 8 | 175 |
| ***Docking between Downregulated common protein domains*** | | | | |
| AnTD-CTF-NFI | -963.8 | 5 | 20 | 199 |
| AnTD-DBS_PH | -866.1 | 2 | 19 | 146 |
| AnTD-NSh2 | -807.1 | 3 | 9 | 147 |
| AnTD-RA+PH | -993.4 | 3 | 16 | 165 |
| AnTD-Sec2p | -1038.0 | 6 | 16 | 159 |
| CTF-NFI-DBS_PH | -737.9 | 5 | 9 | 102 |
| CTF-NFI-Sec2p | -820.1 | 8 | 17 | 181 |
| DBS_PH-NSh2 | -534.8 | 5 | 18 | 153 |
| DBS_PH-RA+PH | -551.4 | 1 | 9 | 87 |
| DBS_PH-Sec2p | -605.4 | 4 | 4 | 96 |
| NSh2-Sec2p | -770.4 | 7 | 12 | 171 |
| RA+PH-Sec2p | -801.4 | 3 | 8 | 114 |
| ***Docking between Upregulated hub protein domains*** | | | | |
| C2IB-Histone H2B | -827.9 | 2 | 10 | 118 |
| C2IB-RPS6E | -609.7 | 9 | 16 | 173 |
| C2IB-A1ARSD | -1055.8 | 2 | 15 | 152 |
| C2IB-CTLLD | -670.7 | 1 | 4 | 303 |
| C2IB-PTRRG2/MT | -689.9 | 5 | 6 | 140 |
| C2IB-ZDM | -740.7 | 2 | 11 | 184 |
| WD40-Histone H2B | -729.4 | 1 | 6 | 94 |
| WD40-RPS6E | -651.0 | 1 | 5 | 84 |
| WD40-A1ARSD | -764.9 | 0 | 6 | 87 |
| WD40- C2IB | -808.2 | 1 | 13 | 123 |
| WD40-PTRRG2/MT | -536.9 | 1 | 3 | 53 |
| WD40-ZDM | -719.4 | 1 | 16 | 123 |
| Histone H2B-RPS6E | -764.6 | 1 | 11 | 137 |
| Histone H2B-A1ARSD | -865.8 | 1 | 4 | 83 |
| Histone H2B-CTLLD | -727.1 | 0 | 10 | 125 |
| Histone H2B-PTRRG2/MT | -788.7 | 3 | 11 | 161 |
| Histone H2B-ZDM | -704.7 | 2 | 9 | 116 |
| RPS6E-CTLLD | -721.9 | 4 | 27 | 150 |
| RPS6E-PTRRG2/MT | -508.8 | 0 | 5 | 69 |
| RPS6E-ZDM | -857.2 | 7 | 23 | 203 |
| A1ARSD-CTLLD | -1175.3 | 0 | 4 | 123 |
| A1ARSD-PTRRG2/MT | -935.5 | 0 | 0 | 5 |
| A1ARSD-ZDM | -1190.8 | 6 | 14 | 152 |
| ***Docking between Upregulated hub protein domains*** | | | | |
| HA_BD-AGC | -678.3 | 3 | 18 | 188 |
| HA_BD-Catalytic domain | -767.5 | 3 | 8 | 186 |
| HA_BD-Rab11 | -672.1 | 6 | 18 | 160 |
| HA_BD-Esp15 | -702.3 | 4 | 14 | 143 |
| HA_BD-WD40 | -760.1 | 0 | 9 | 112 |
| HA_BD-RhoGAp | -590.0 | 4 | 11 | 147 |
| HA_BD-PH | -747.2 | 2 | 13 | 136 |
| FOXP-AGC | -757.7 | 6 | 17 | 161 |
| FOXP-Catalytic | -770.4 | 6 | 19 | 183 |
| FOXP- Rab11 | -609.3 | 9 | 23 | 187 |
| FOXP-Esp15 | -652.7 | 2 | 16 | 135 |
| FOXP-WD40 | -956.6 | 4 | 21 | 218 |
| FOXP-RhoGAp | -693.7 | 2 | 8 | 110 |
| FOXP-PH | -794.6 | 7 | 29 | 255 |
| TAS2RS14-AGC | -1073.4 | 2 | 8 | 154 |
| TAS2RS14-Catalytic | -1095.3 | 0 | 4 | 94 |
| TAS2RS14-Rab11 | -914.5 | 1 | 4 | 100 |
| TAS2RS14-Esp15 | -927.2 | 1 | 6 | 109 |
| TAS2RS14-WD40 | -1343.8 | 3 | 9 | 152 |
| TAS2RS14-RhoGAp | -1195.1 | 2 | 4 | 147 |
| TAS2RS14-PH | -1052.5 | 2 | 13 | 118 |
| AGC-Rab11 | -707.2 | 2 | 5 | 126 |
| AGC-Esp15 | -643.7 | 1 | 11 | 201 |
| AGC-WD40 | -682.9 | 1 | 8 | 162 |
| AGC-RhoGAp | -575.0 | 3 | 10 | 127 |
| AGC-PH | -996.5 | 4 | 17 | 175 |
| Catalytic-Rab11 | -667.9 | 3 | 3 | 372 |
| Catalytic-Esp15 | -679.9 | 1 | 13 | 167 |
| Catalytic-WD40 | -589.3 | 2 | 17 | 162 |
| Catalytic-RhoGAp | -656.4 | 2 | 17 | 162 |
| Catalytic-PH | -856.9 | 4 | 9 | 154 |
| Rab11-RhoGAp | -639.2 | 3 | 9 | 135 |
| Rab11-PH | -766.1 | 1 | 11 | 124 |
| Esp15-RhoGAp | -582.1 | 1 | 6 | 116 |
| Esp15-PH | -800.7 | 2 | 10 | 170 |
| WD40-RhoGAp | -591.0 | 2 | 12 | 140 |
| WD40-PH | -908.5 | 3 | 6 | 124 |

**Supplementary table 2**: The docking energies, number of formed hydrogen bonds, salt bridges and non-bonded interactions of the 73 docked domain complexes.


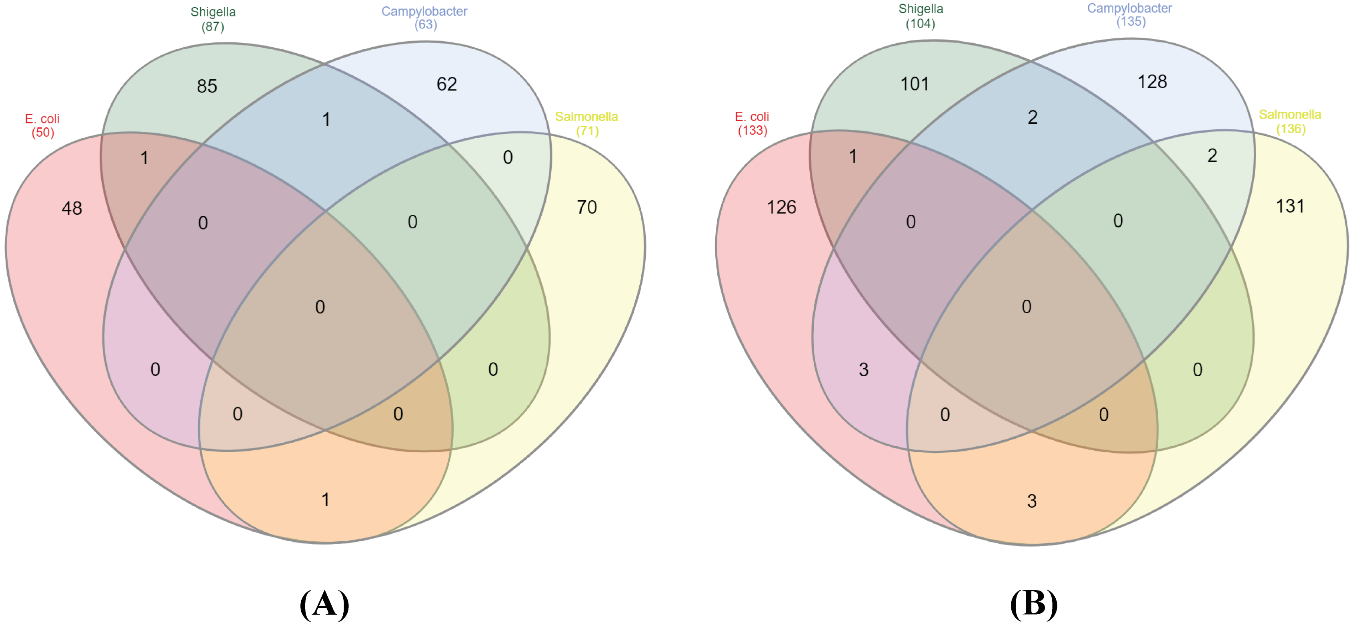


**Supplementary Figure 1**: Number of common upregulated and downregulated genes measured from Interactivenn server.


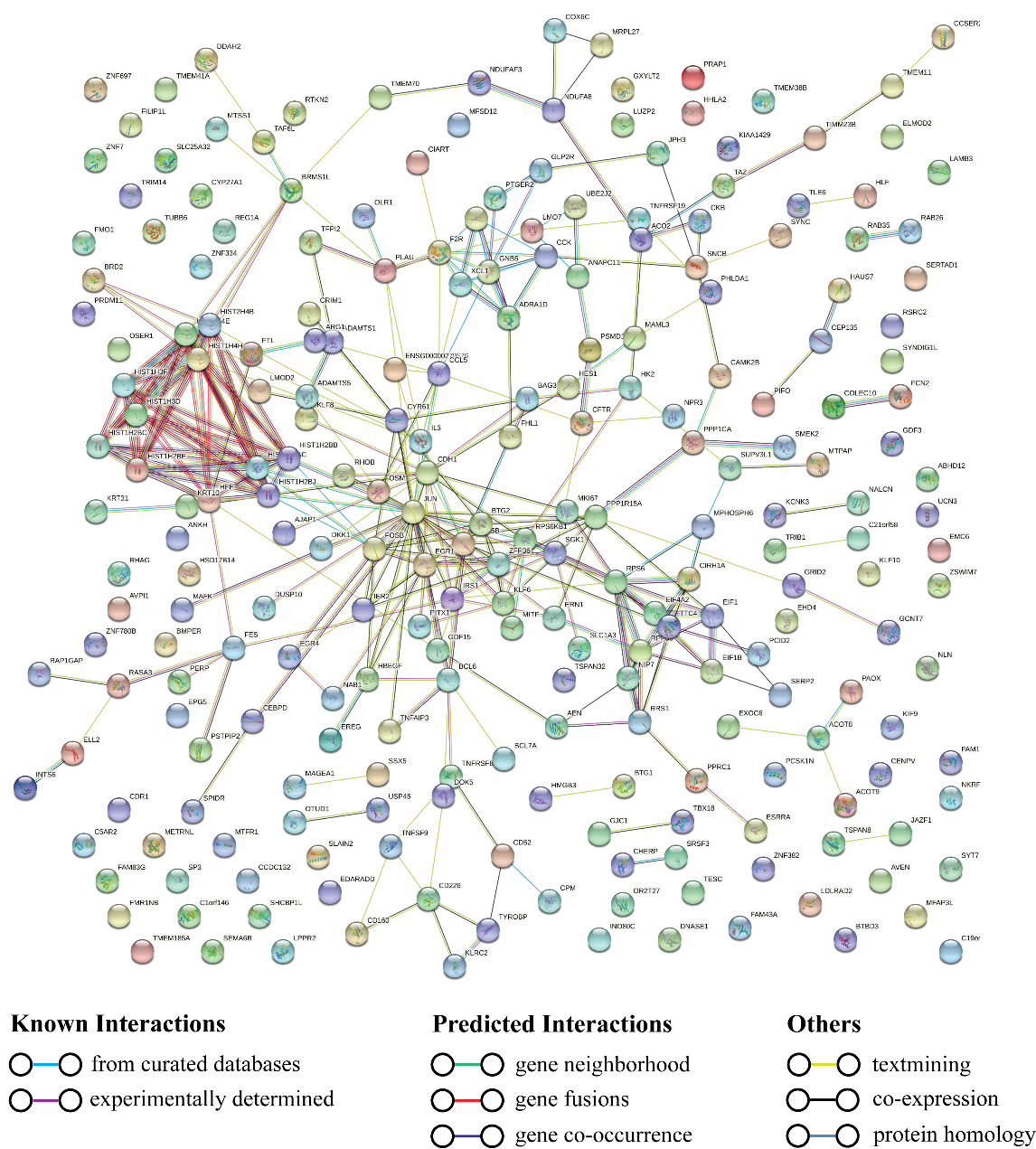


**Supplementary Figure 2**: STRING protein-protein interaction network of upregulated genes. Here, circles represent genes while lines represent the interaction of proteins between genes.


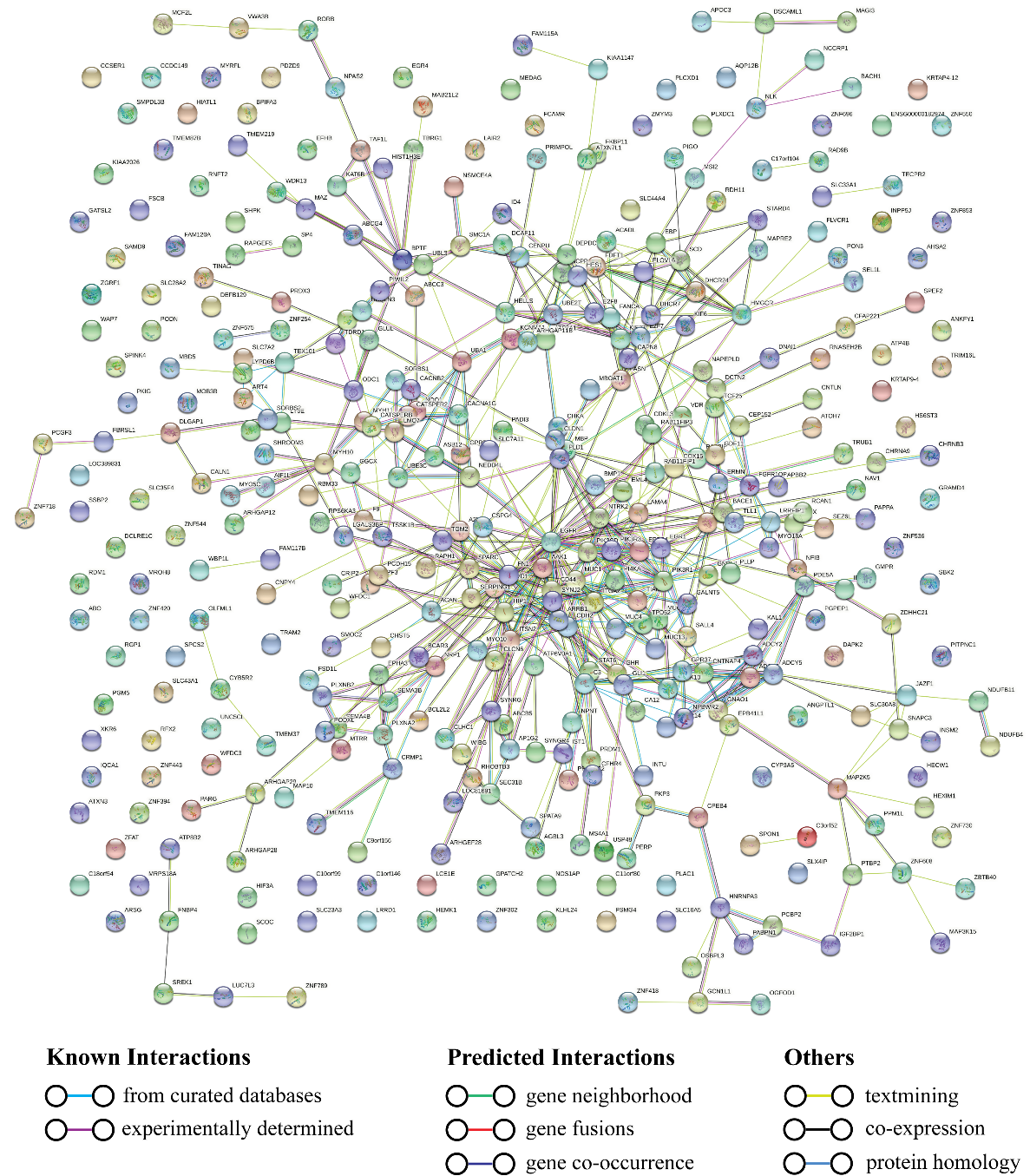


**Supplementary Figure 3**: STRING protein-protein interaction network of downregulated genes. Here, circles represent genes while lines represent the interaction of proteins between genes.


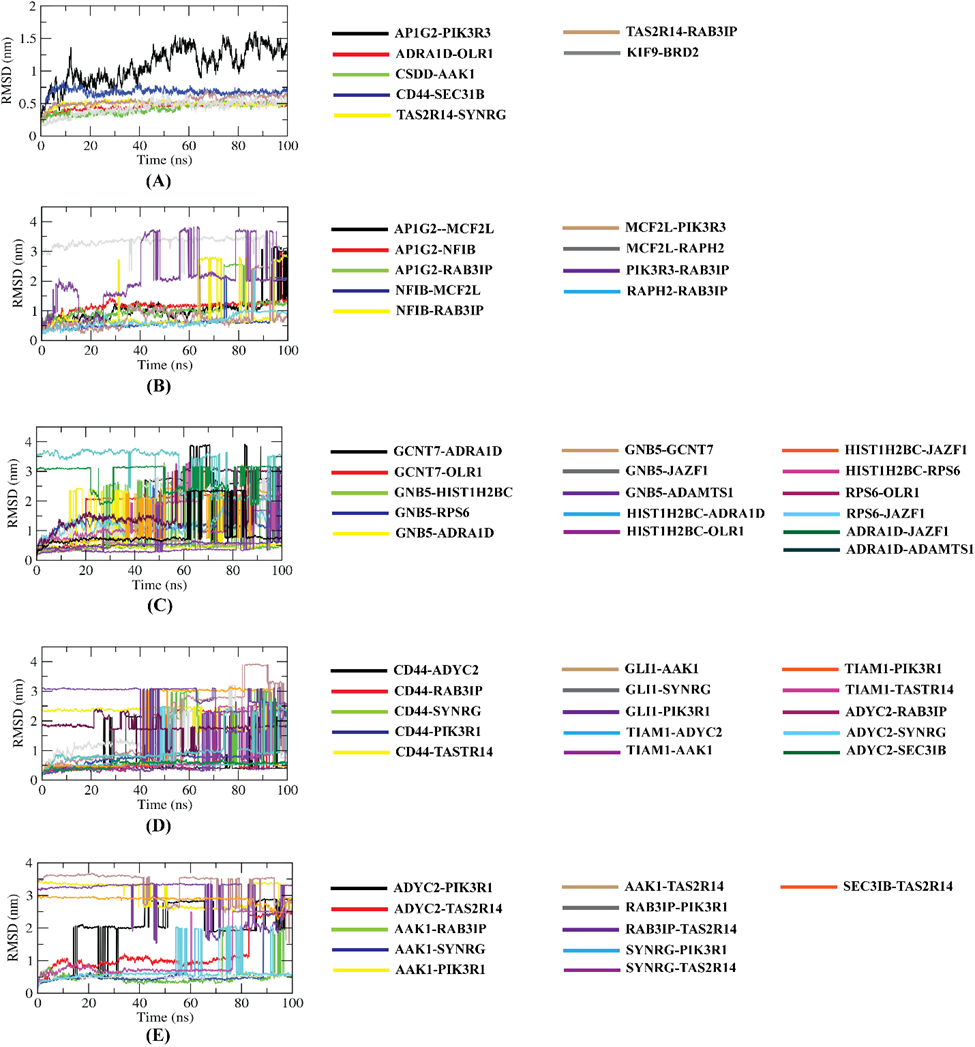


**Supplementary Figure 4**: The RMSD analysis of the 7 stable complexes (A) and 53 unstable complexes (B-E).
