# Supplementary material for: Pathogen-driven gene expression patterns lead to a novel approach to the identification of common therapeutic targets
Source: Sci Rep. 2022 Dec 6;12:21070. doi: 10.1038/s41598-022-25102-8 (PMC9726901; doi:10.1038/s41598-022-25102-8)
Supplement: Supplementary file 2 — Supplementary Information 2. [file 41598_2022_25102_MOESM2_ESM.docx]

**Supplementary movie 1**: Simulation movie of AnTD-nSH2 complex.

**Supplementary movie 2**: Simulation movie of AnTD-RA_PH complex.

**Supplementary movie 3**: Simulation movie of RA_PH-Dbs_PH complex.

**Supplementary movie 4**: Simulation movie of C2IBe-H2B complex.

**Supplementary movie 5**: Simulation movie of C2IBe-PTRRG2MT complex.

**Supplementary movie 6**: Simulation movie of C2IBe-RPS6e complex.

**Supplementary movie 7**: Simulation movie of C2IBe-ZDM complex.

**Supplementary movie 8**: Simulation movie of ZDM-H2B complex.

**Supplementary movie 9**: Simulation movie of ZDM-RPS6e complex.

**Supplementary movie 10**: Simulation movie of FOXP-AGC complex.

**Supplementary movie 11**: Simulation movie of FOXP-PH complex.

**Supplementary movie 12**: Simulation movie of FOXP-Rab11 complex.

**Supplementary movie 13**: Simulation movie of WD40-Catalytic_domain complex.

**Supplementary movie 14**: Simulation movie of WD40-RhoGAP complex.

**Supplementary movie 15**: Simulation movie of WD40-TAS2R complex.
