# Supplementary material for: Pathogen-driven gene expression patterns lead to a novel approach to the identification of common therapeutic targets
Source: Sci Rep. 2022 Dec 6;12:21070. doi: 10.1038/s41598-022-25102-8 (PMC9726901; doi:10.1038/s41598-022-25102-8)
Supplement: Supplementary file 9 — Supplementary Information 9. [file 41598_2022_25102_MOESM9_ESM.pdf]

PROCHECK

Ramachandran Plot

saves

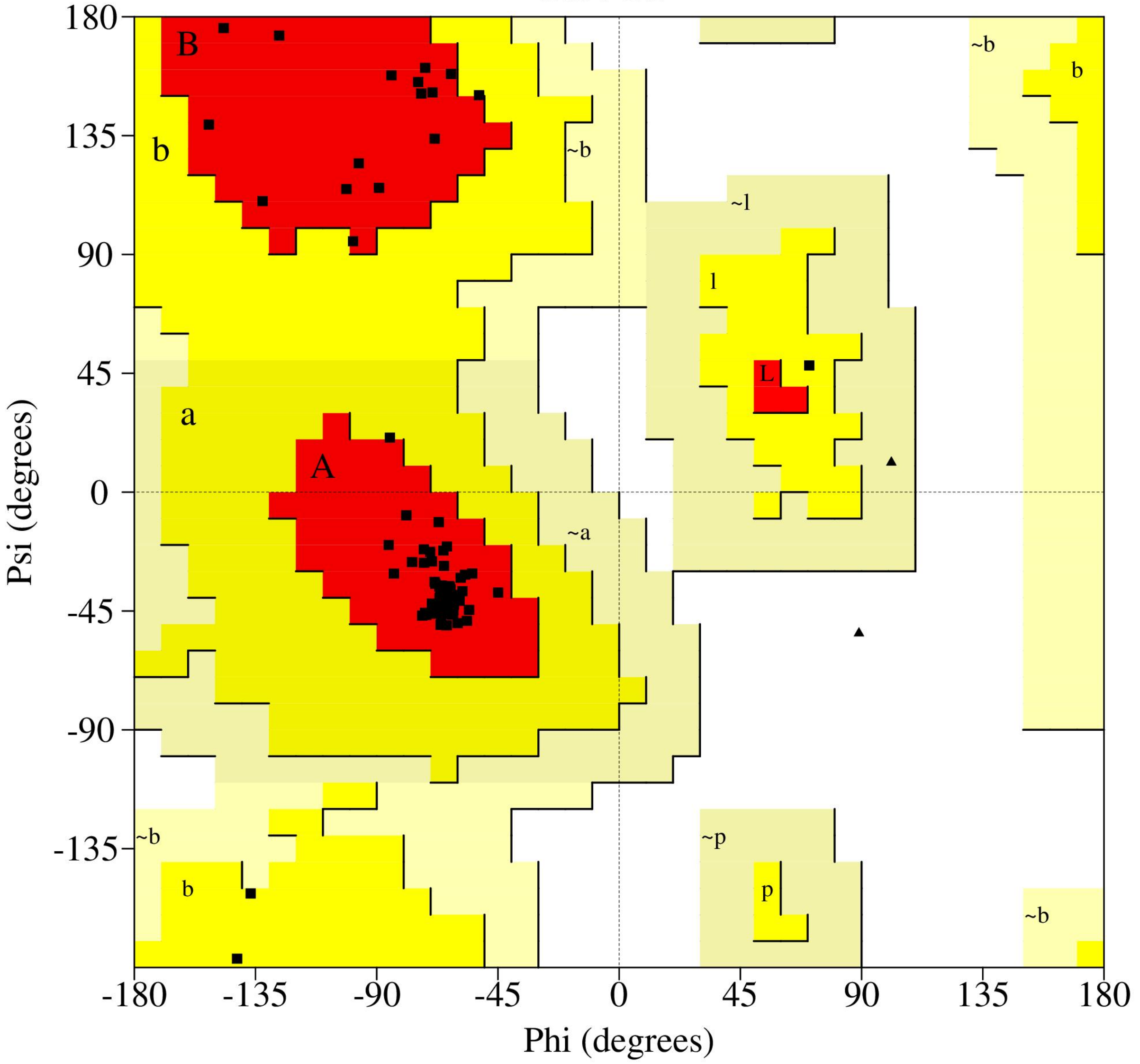

Plot statistics

Residues in most favoured regions [A,B,L]	88	94.6%
Residues in additional allowed regions [a,b,l,p]	5	5.4%
Residues in generously allowed regions [~a,~b,~l,~p]	0	0.0%
Residues in disallowed regions	0	0.0%
	----	------
Number of non-glycine and non-proline residues	93	100.0%
Number of end-residues (excl. Gly and Pro)	2	
Number of glycine residues (shown as triangles)	2	
Number of proline residues	5	

Total number of residues	102	

Based on an analysis of 118 structures of resolution of at least 2.0 Angstroms and R-factor no greater than 20%, a good quality model would be expected to have over 90% in the most favoured regions.

PROCHECK

Ramachandran Plot

saves

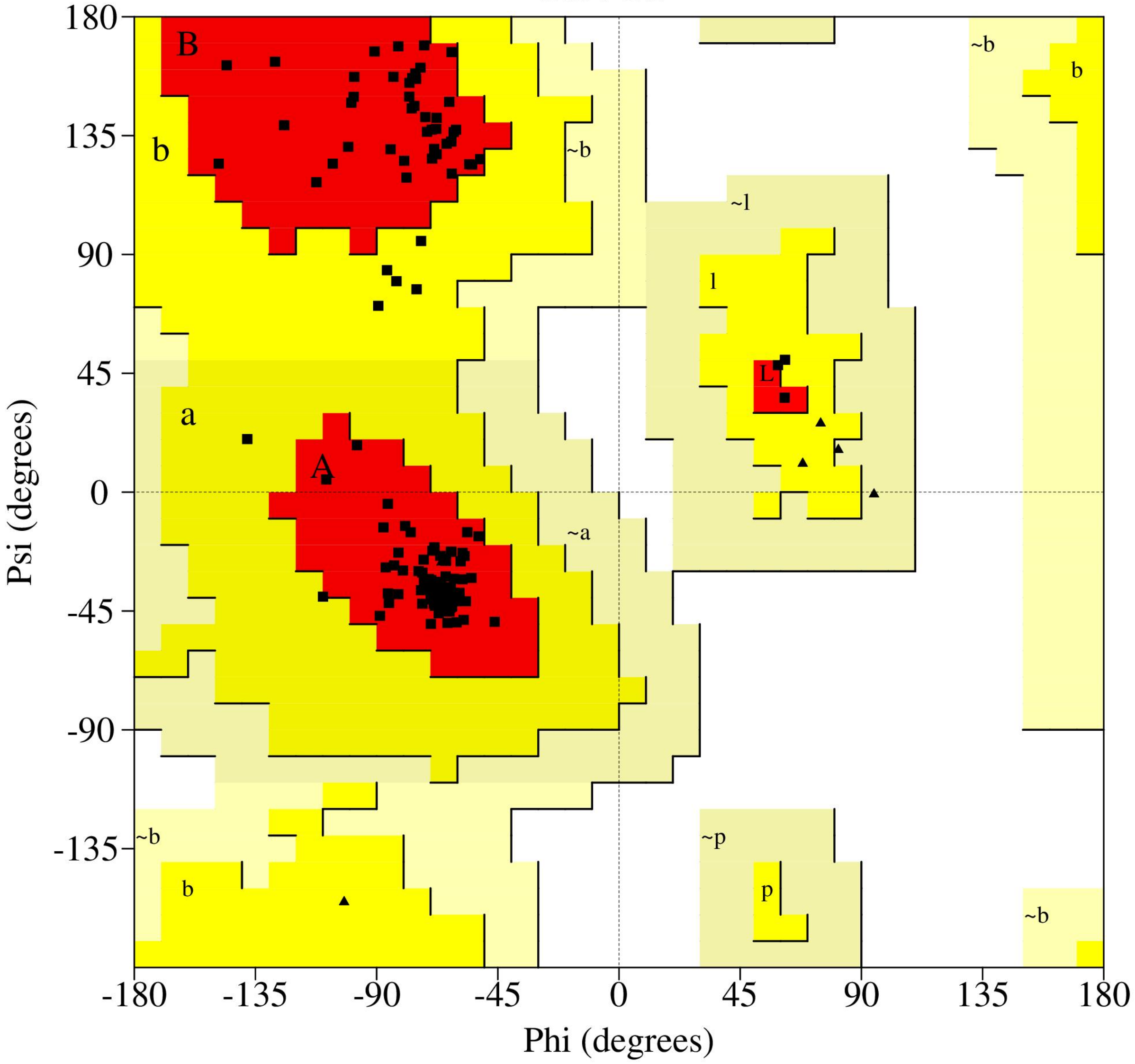

Plot statistics

Residues in most favoured regions [A,B,L]	169	96.0%
Residues in additional allowed regions [a,b,l,p]	7	4.0%
Residues in generously allowed regions [~a,~b,~l,~p]	0	0.0%
Residues in disallowed regions	0	0.0%
	----	------
Number of non-glycine and non-proline residues	176	100.0%
Number of end-residues (excl. Gly and Pro)	1	
Number of glycine residues (shown as triangles)	6	
Number of proline residues	11	

Total number of residues	194	

Based on an analysis of 118 structures of resolution of at least 2.0 Angstroms and R-factor no greater than 20%, a good quality model would be expected to have over 90% in the most favoured regions.

PROCHECK

Ramachandran Plot

saves

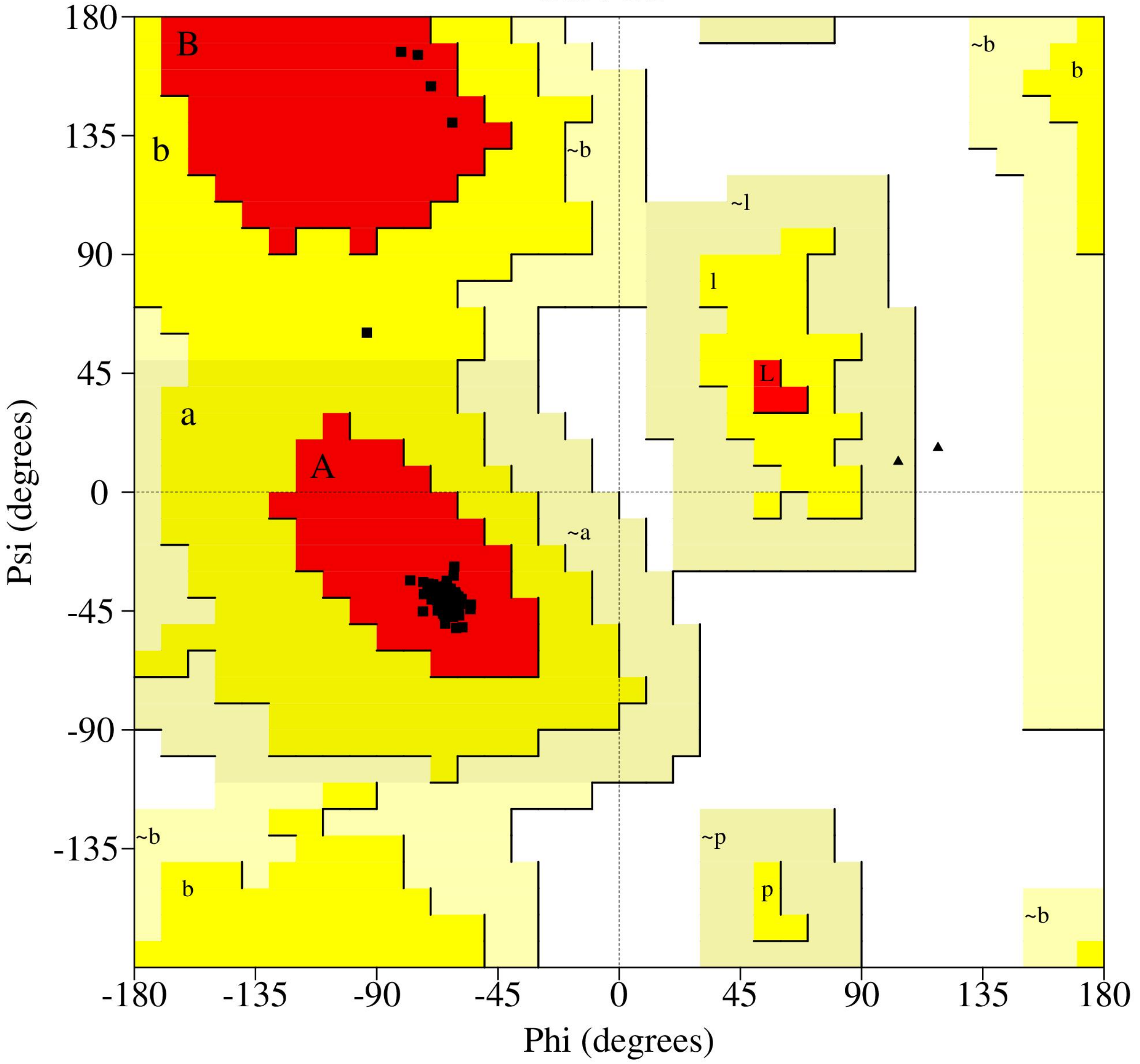

Plot statistics

Residues in most favoured regions [A,B,L]	160	99.4%
Residues in additional allowed regions [a,b,l,p]	1	0.6%
Residues in generously allowed regions [~a,~b,~l,~p]	0	0.0%
Residues in disallowed regions	0	0.0%
	----	------
Number of non-glycine and non-proline residues	161	100.0%
Number of end-residues (excl. Gly and Pro)	2	
Number of glycine residues (shown as triangles)	4	
Number of proline residues	1	

Total number of residues	168	

Based on an analysis of 118 structures of resolution of at least 2.0 Angstroms and R-factor no greater than 20%, a good quality model would be expected to have over 90% in the most favoured regions.

PROCHECK

Ramachandran Plot

saves

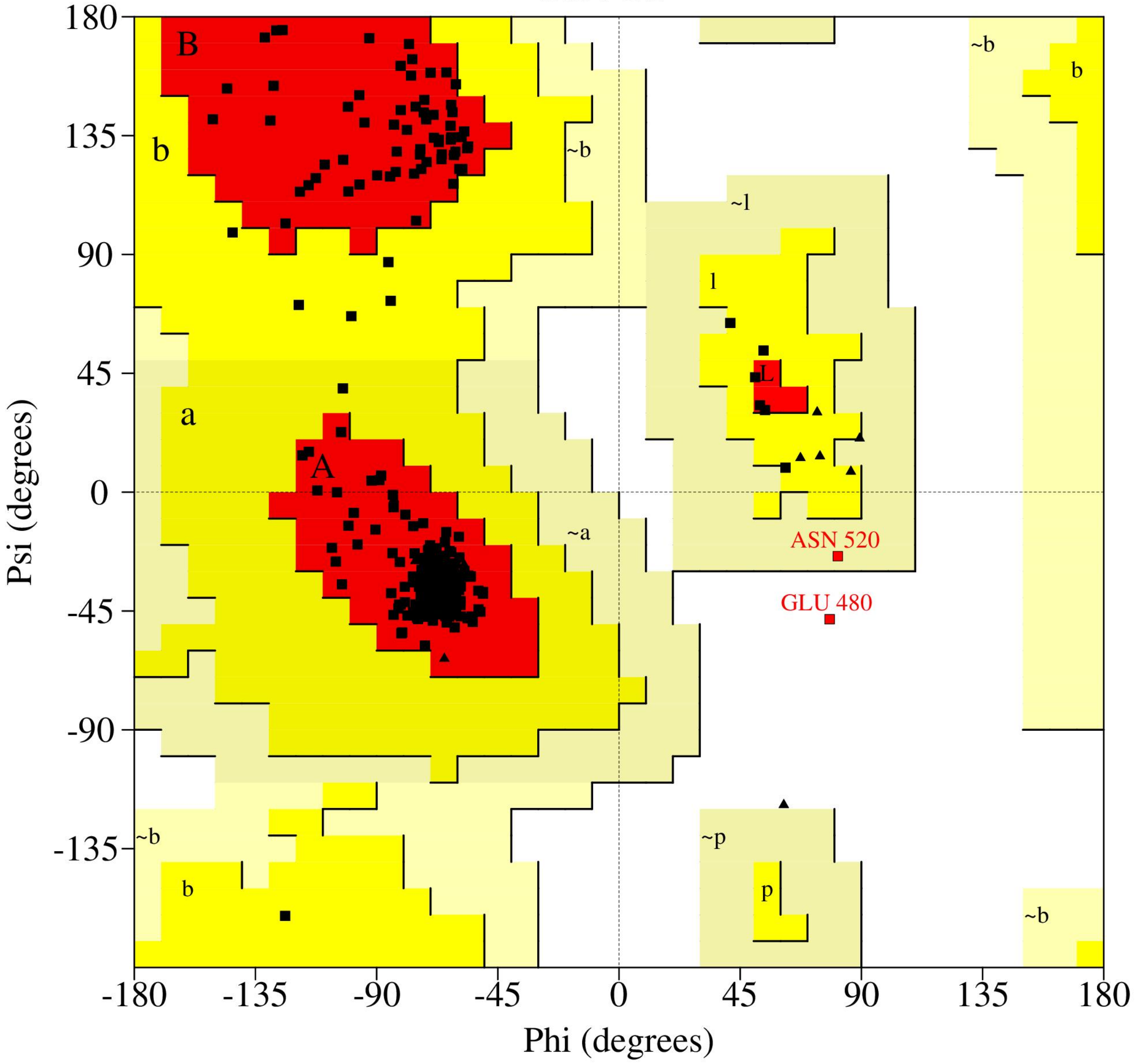

Plot statistics

Residues in most favoured regions [A,B,L]	492	97.6%
Residues in additional allowed regions [a,b,l,p]	10	2.0%
Residues in generously allowed regions [~a,~b,~l,~p]	1	0.2%
Residues in disallowed regions	1	0.2%
	----	------
Number of non-glycine and non-proline residues	504	100.0%
Number of end-residues (excl. Gly and Pro)	2	
Number of glycine residues (shown as triangles)	26	
Number of proline residues	19	

Total number of residues	551	

Based on an analysis of 118 structures of resolution of at least 2.0 Angstroms and R-factor no greater than 20%, a good quality model would be expected to have over 90% in the most favoured regions.

PROCHECK

Ramachandran Plot

saves

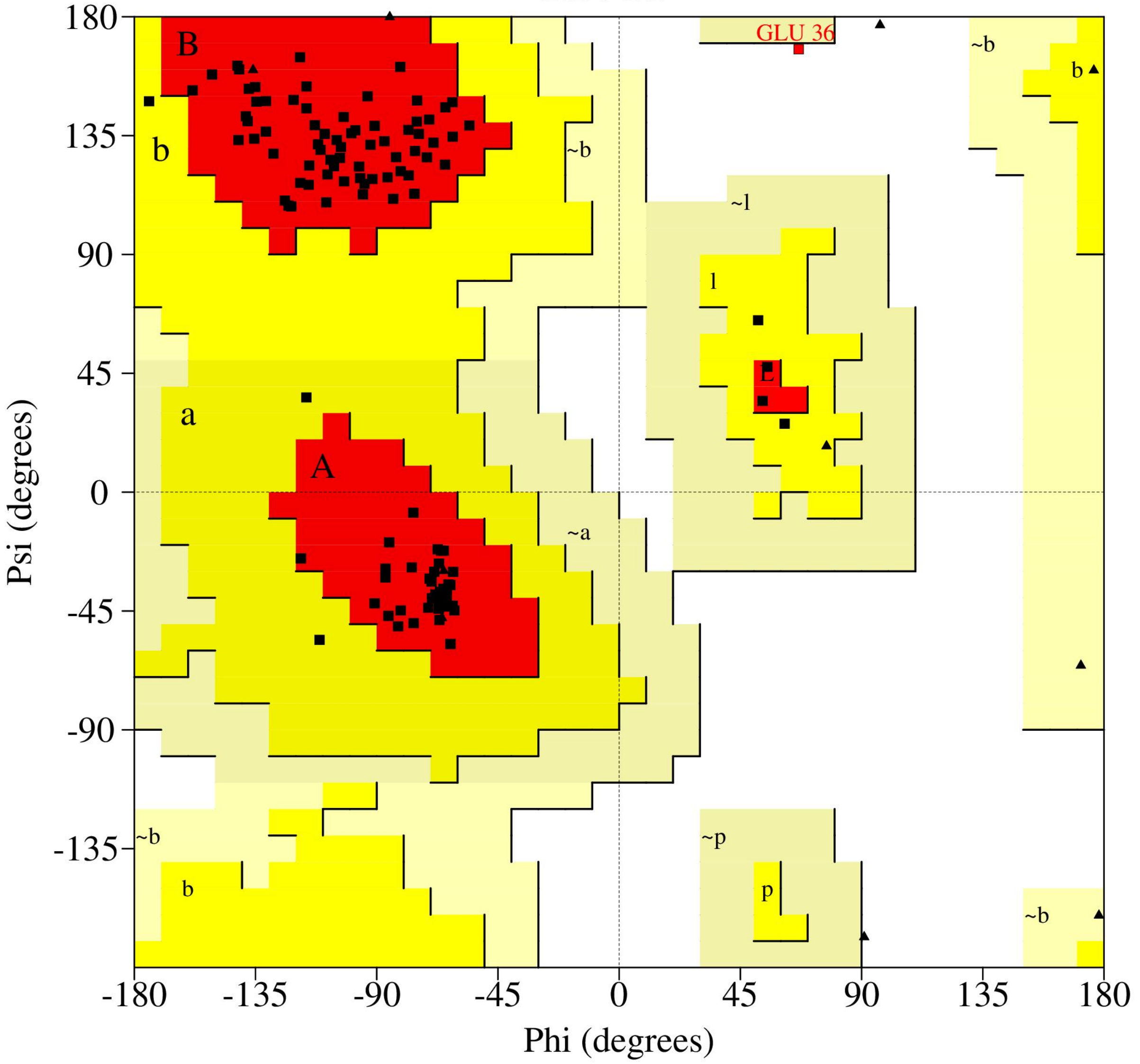

Plot statistics

Residues in most favoured regions [A,B,L]	110	94.8%
Residues in additional allowed regions [a,b,l,p]	5	4.3%
Residues in generously allowed regions [~a,~b,~l,~p]	0	0.0%
Residues in disallowed regions	1	0.9%
	----	------
Number of non-glycine and non-proline residues	116	100.0%
Number of end-residues (excl. Gly and Pro)	2	
Number of glycine residues (shown as triangles)	10	
Number of proline residues	4	

Total number of residues	132	

Based on an analysis of 118 structures of resolution of at least 2.0 Angstroms and R-factor no greater than 20%, a good quality model would be expected to have over 90% in the most favoured regions.

PROCHECK

Ramachandran Plot

saves

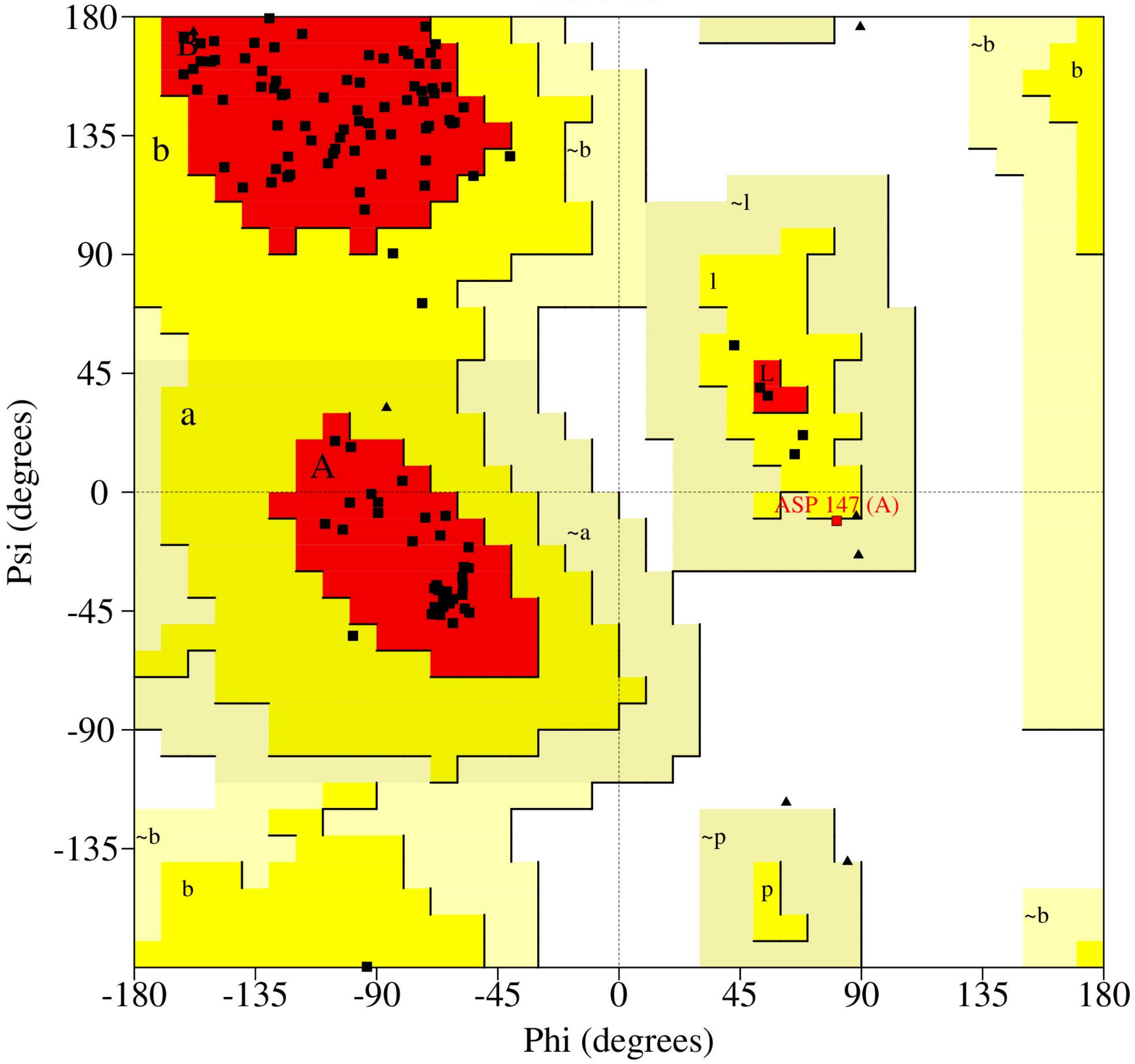

Plot statistics

Residues in most favoured regions [A,B,L]	105	91.3%
Residues in additional allowed regions [a,b,l,p]	9	7.8%
Residues in generously allowed regions [~a,~b,~l,~p]	1	0.9%
Residues in disallowed regions	0	0.0%
	----	------
Number of non-glycine and non-proline residues	115	100.0%
Number of end-residues (excl. Gly and Pro)	2	
Number of glycine residues (shown as triangles)	7	
Number of proline residues	8	

Total number of residues	132	

Based on an analysis of 118 structures of resolution of at least 2.0 Angstroms and R-factor no greater than 20%, a good quality model would be expected to have over 90% in the most favoured regions.

saves_01.ps

PROCHECK

Ramachandran Plot

saves

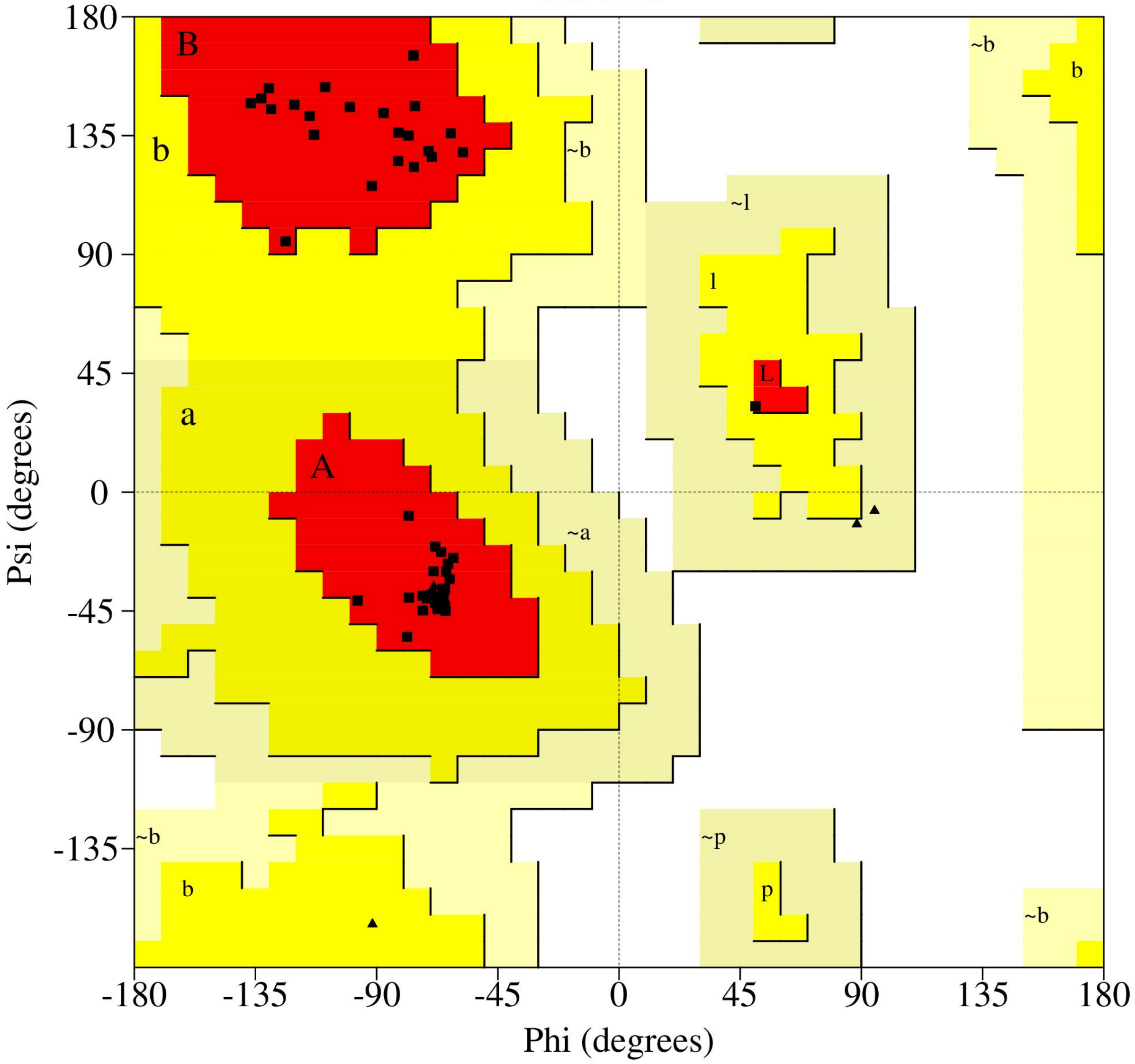

Plot statistics

Residues in most favoured regions [A,B,L]	49	100.0%
Residues in additional allowed regions [a,b,l,p]	0	0.0%
Residues in generously allowed regions [~a,~b,~l,~p]	0	0.0%
Residues in disallowed regions	0	0.0%
	----	------
Number of non-glycine and non-proline residues	49	100.0%
Number of end-residues (excl. Gly and Pro)	1	
Number of glycine residues (shown as triangles)	7	
Number of proline residues	4	

Total number of residues	61	

Based on an analysis of 118 structures of resolution of at least 2.0 Angstroms and R-factor no greater than 20%, a good quality model would be expected to have over 90% in the most favoured regions.

saves_01.ps

PROCHECK

Ramachandran Plot

saves

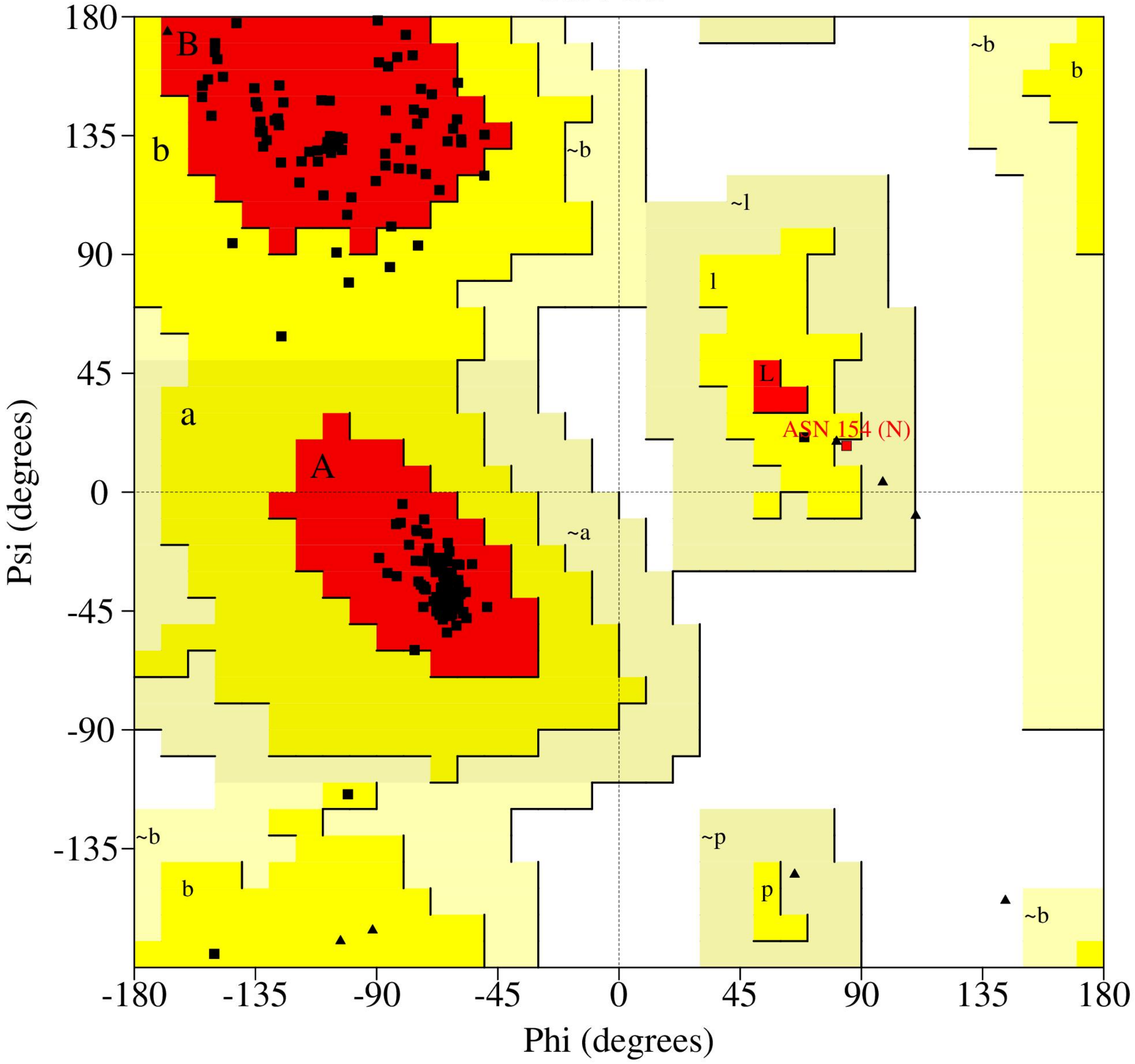

Plot statistics

Residues in most favoured regions [A,B,L]	172	93.5%
Residues in additional allowed regions [a,b,l,p]	11	6.0%
Residues in generously allowed regions [~a,~b,~l,~p]	1	0.5%
Residues in disallowed regions	0	0.0%
	----	------
Number of non-glycine and non-proline residues	184	100.0%
Number of end-residues (excl. Gly and Pro)	2	
Number of glycine residues (shown as triangles)	9	
Number of proline residues	8	

Total number of residues	203	

Based on an analysis of 118 structures of resolution of at least 2.0 Angstroms and R-factor no greater than 20%, a good quality model would be expected to have over 90% in the most favoured regions.

PROCHECK

Ramachandran Plot

saves

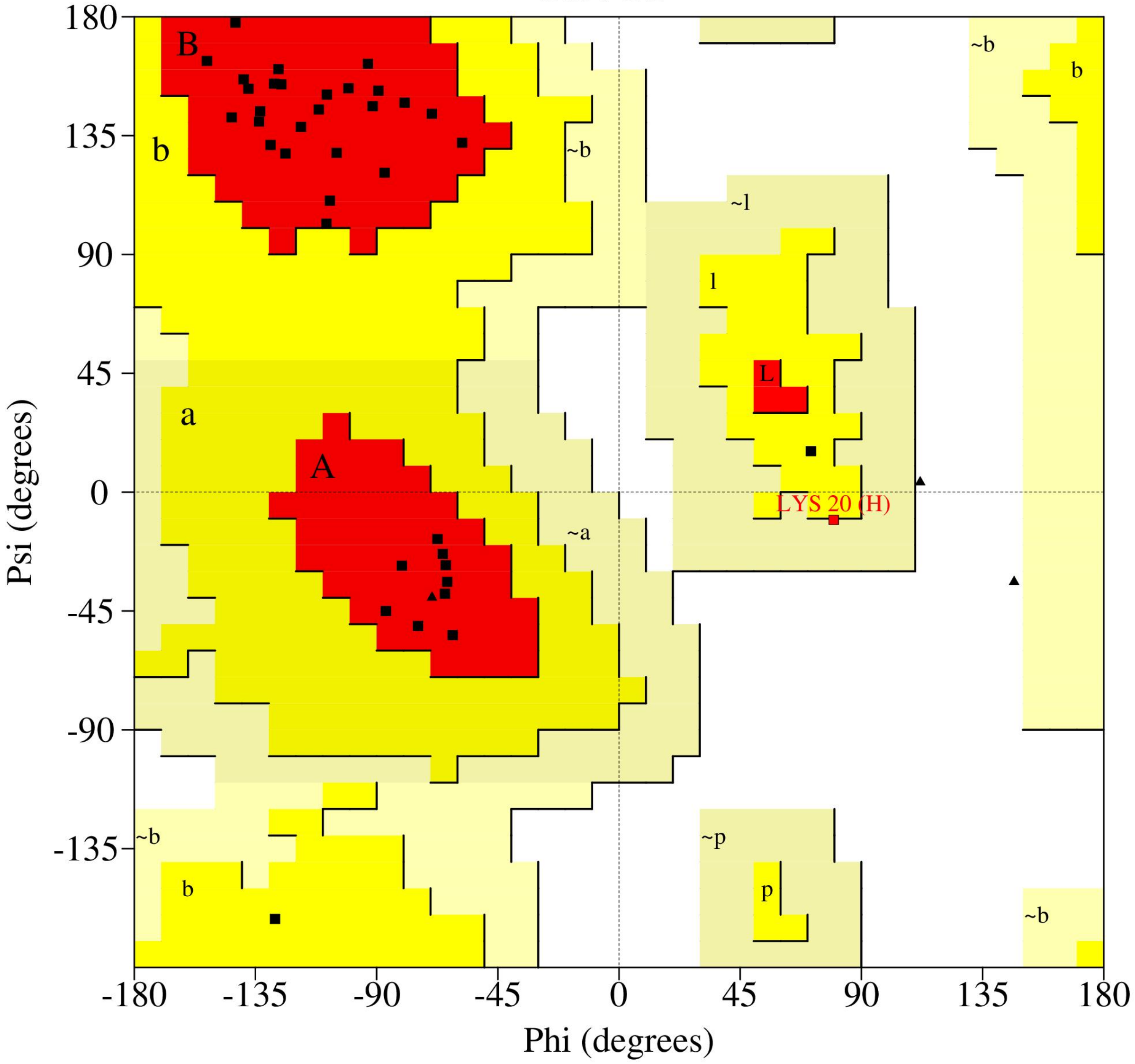

Plot statistics

Residues in most favoured regions [A,B,L]	35	92.1%
Residues in additional allowed regions [a,b,l,p]	2	5.3%
Residues in generously allowed regions [~a,~b,~l,~p]	1	2.6%
Residues in disallowed regions	0	0.0%
	----	------
Number of non-glycine and non-proline residues	38	100.0%
Number of end-residues (excl. Gly and Pro)	2	
Number of glycine residues (shown as triangles)	3	
Number of proline residues	0	

Total number of residues	43	

Based on an analysis of 118 structures of resolution of at least 2.0 Angstroms and R-factor no greater than 20%, a good quality model would be expected to have over 90% in the most favoured regions.

PROCHECK

Ramachandran Plot

saves

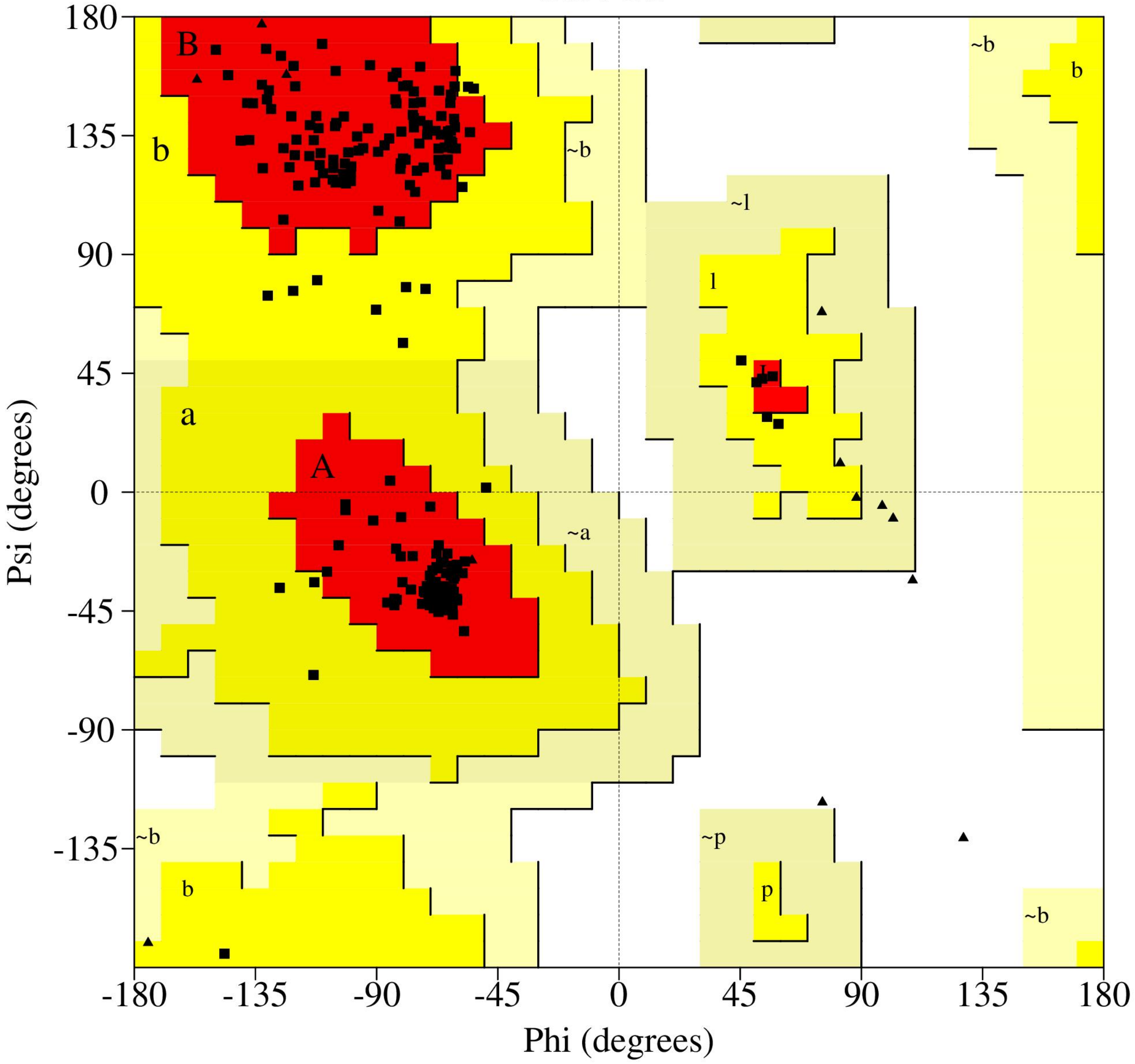

Plot statistics

Residues in most favoured regions [A,B,L]	216	93.5%
Residues in additional allowed regions [a,b,l,p]	15	6.5%
Residues in generously allowed regions [~a,~b,~l,~p]	0	0.0%
Residues in disallowed regions	0	0.0%
	----	------
Number of non-glycine and non-proline residues	231	100.0%
Number of end-residues (excl. Gly and Pro)	2	
Number of glycine residues (shown as triangles)	13	
Number of proline residues	14	

Total number of residues	260	

Based on an analysis of 118 structures of resolution of at least 2.0 Angstroms and R-factor no greater than 20%, a good quality model would be expected to have over 90% in the most favoured regions.

saves_01.ps

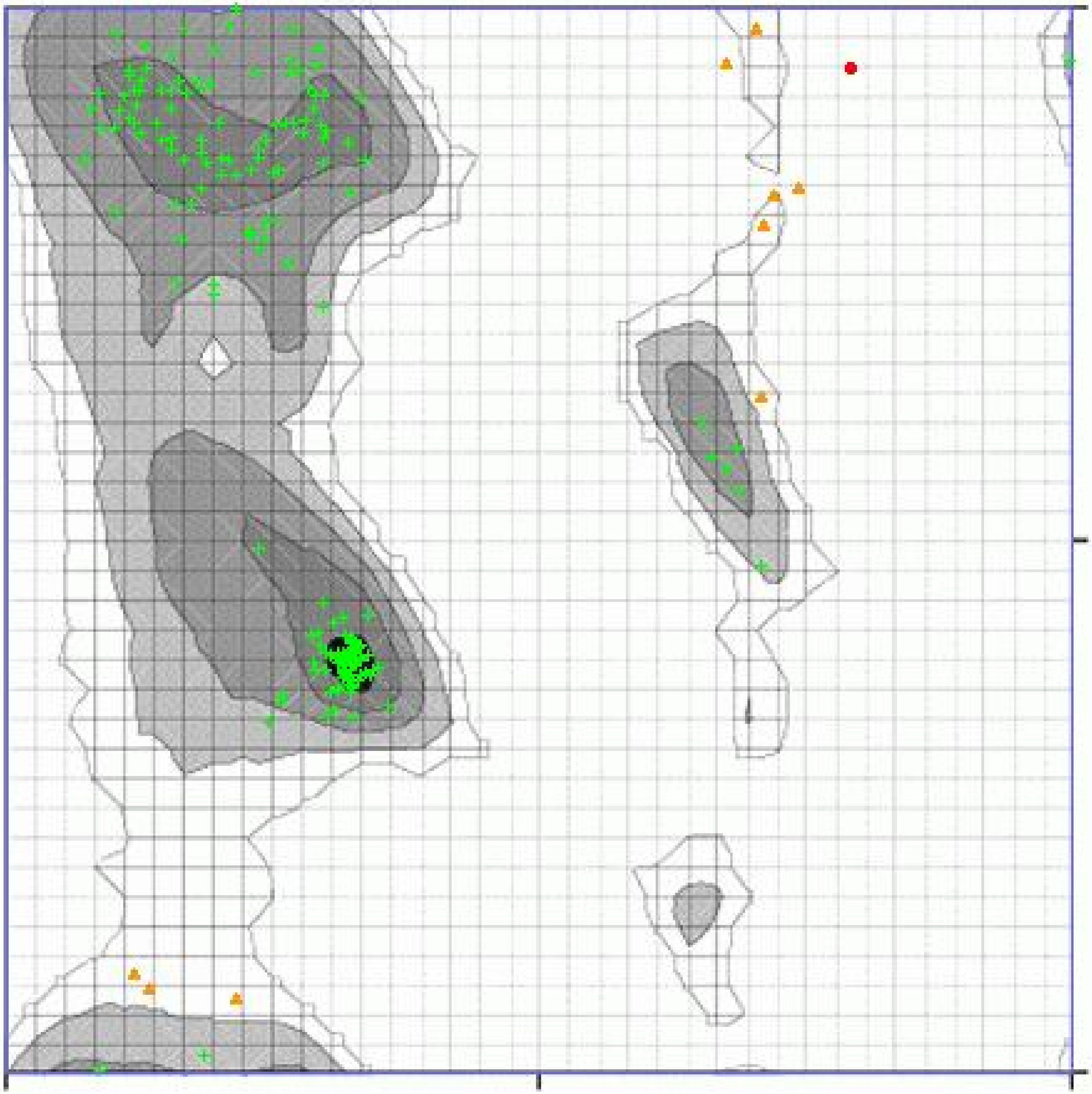

PROCHECK

Ramachandran Plot

saves

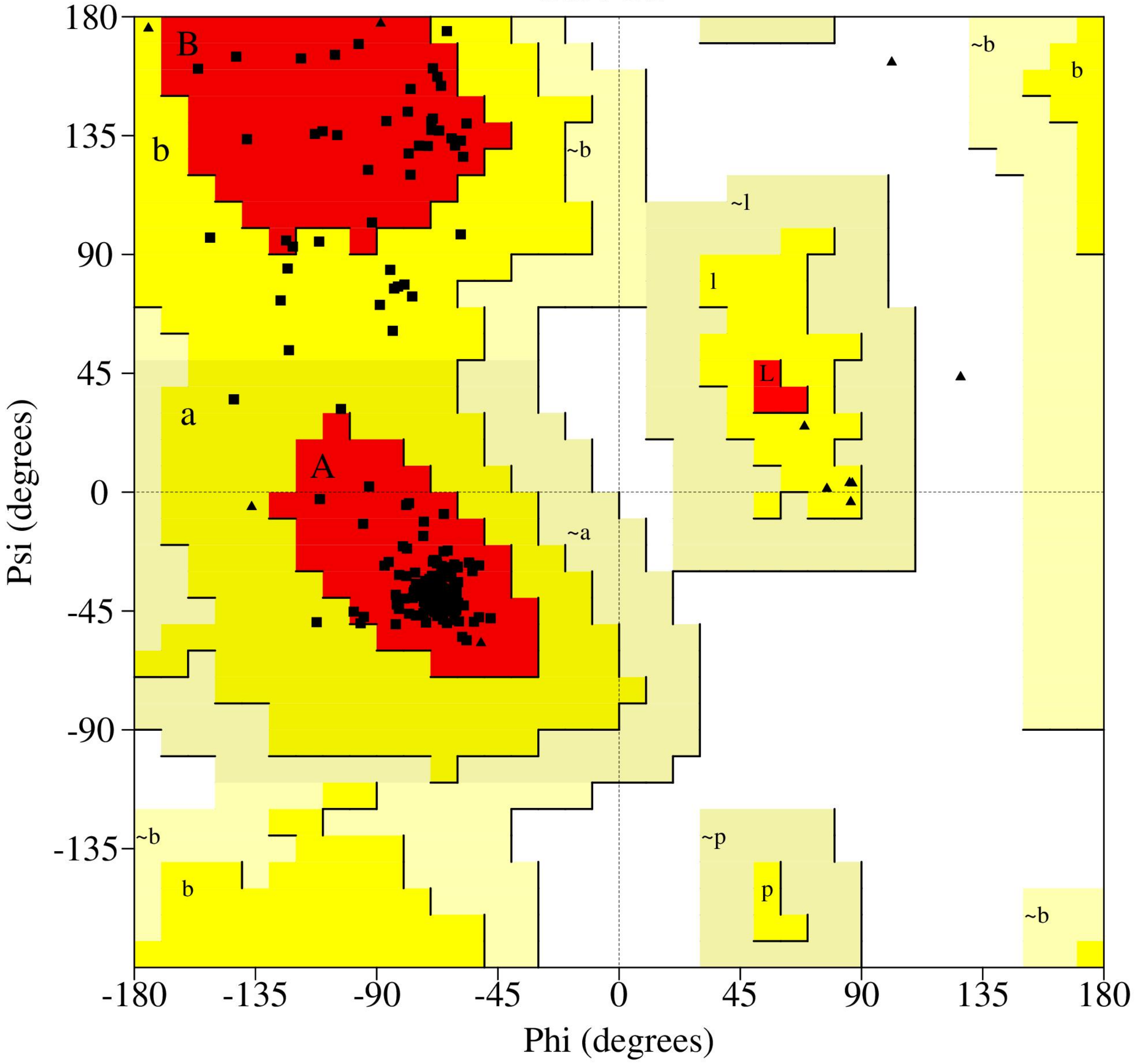

Plot statistics

Residues in most favoured regions [A,B,L]	261	94.2%
Residues in additional allowed regions [a,b,l,p]	16	5.8%
Residues in generously allowed regions [~a,~b,~l,~p]	0	0.0%
Residues in disallowed regions	0	0.0%
	----	------
Number of non-glycine and non-proline residues	277	100.0%
Number of end-residues (excl. Gly and Pro)	1	
Number of glycine residues (shown as triangles)	22	
Number of proline residues	13	

Total number of residues	313	

Based on an analysis of 118 structures of resolution of at least 2.0 Angstroms and R-factor no greater than 20%, a good quality model would be expected to have over 90% in the most favoured regions.

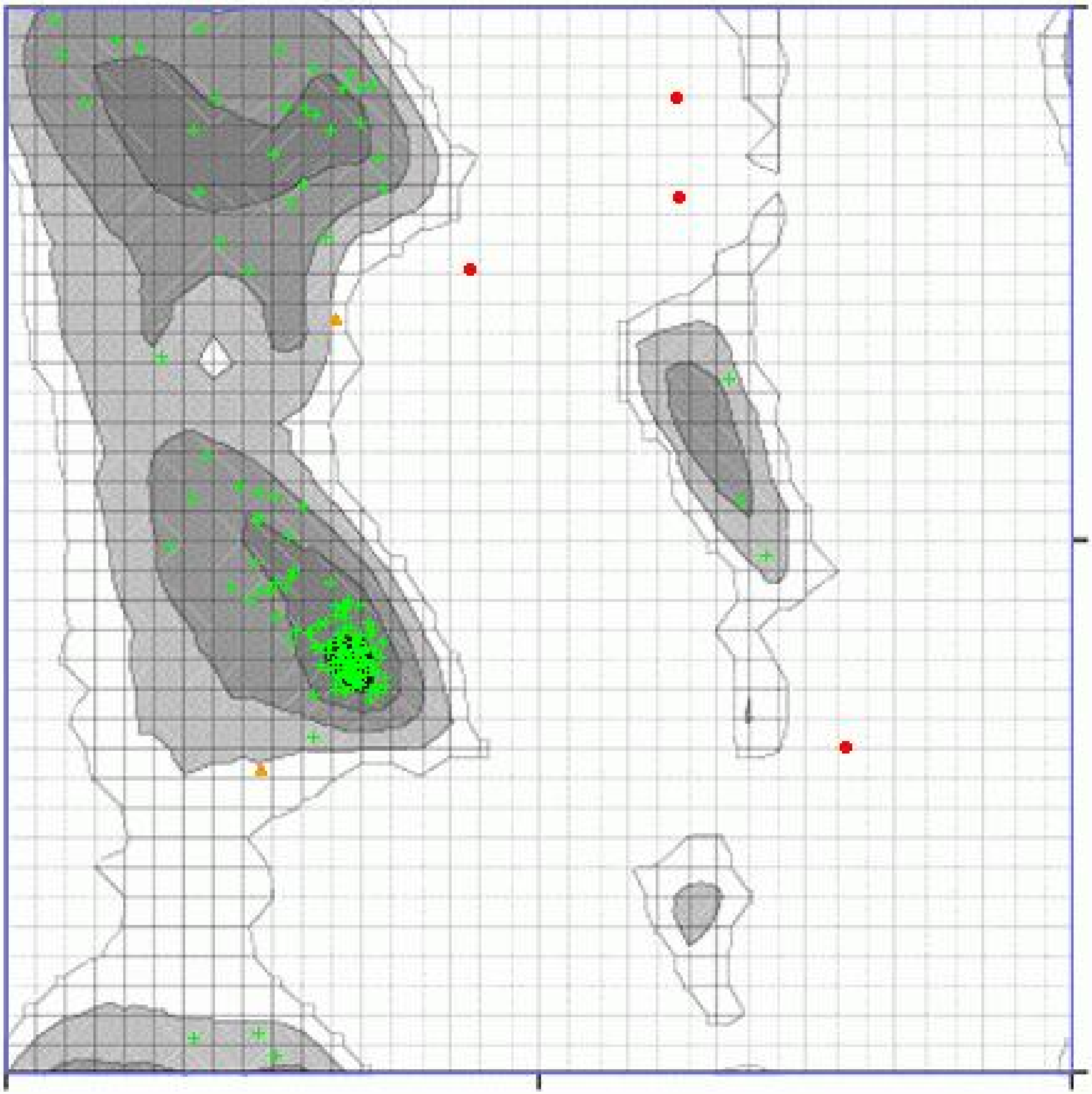

PROCHECK

Ramachandran Plot

saves

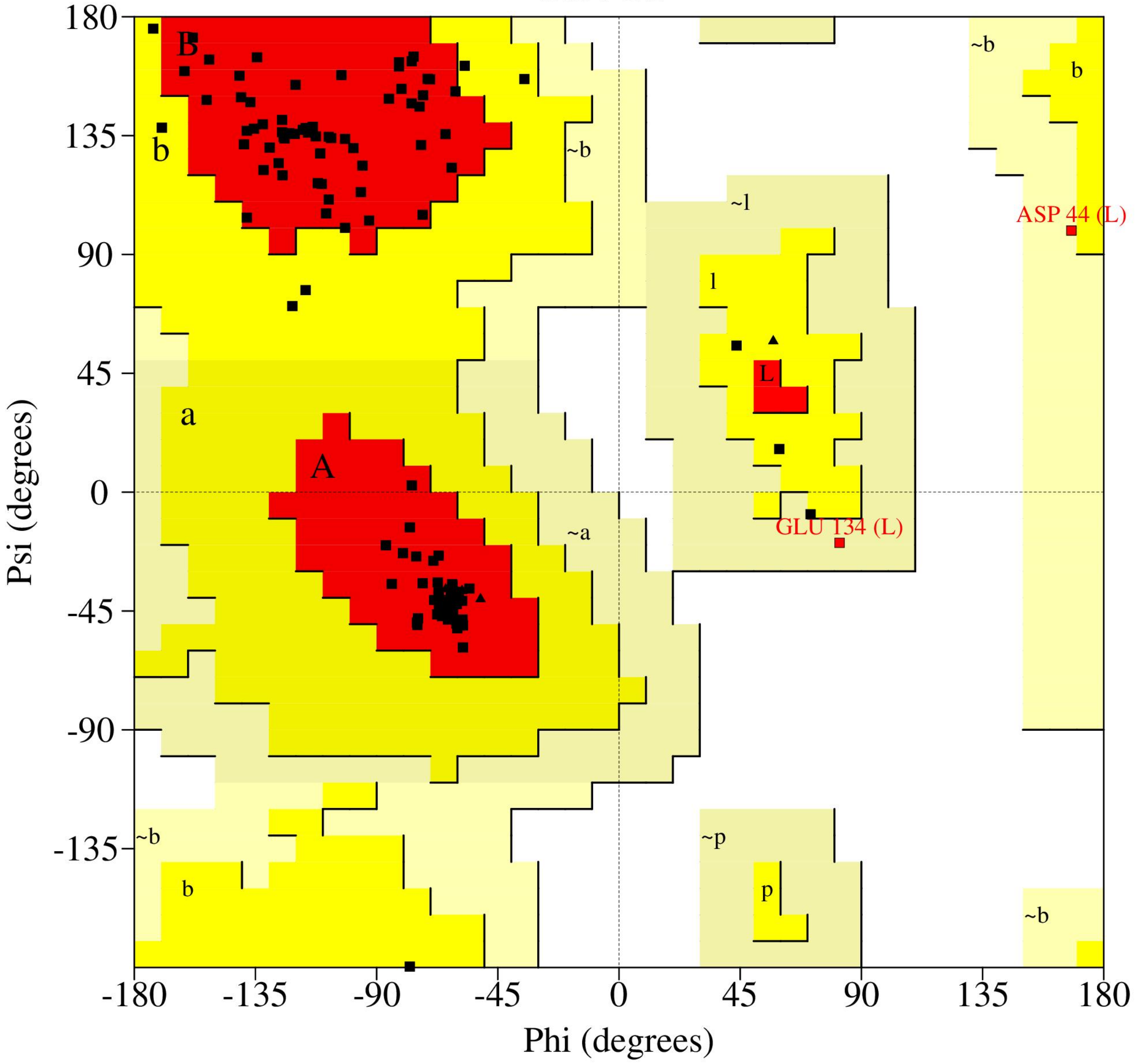

Plot statistics

Residues in most favoured regions [A,B,L]	112	91.1%
Residues in additional allowed regions [a,b,l,p]	9	7.3%
Residues in generously allowed regions [~a,~b,~l,~p]	2	1.6%
Residues in disallowed regions	0	0.0%
	----	------
Number of non-glycine and non-proline residues	123	100.0%
Number of end-residues (excl. Gly and Pro)	7	
Number of glycine residues (shown as triangles)	4	
Number of proline residues	6	

Total number of residues	140	

Based on an analysis of 118 structures of resolution of at least 2.0 Angstroms and R-factor no greater than 20%, a good quality model would be expected to have over 90% in the most favoured regions.

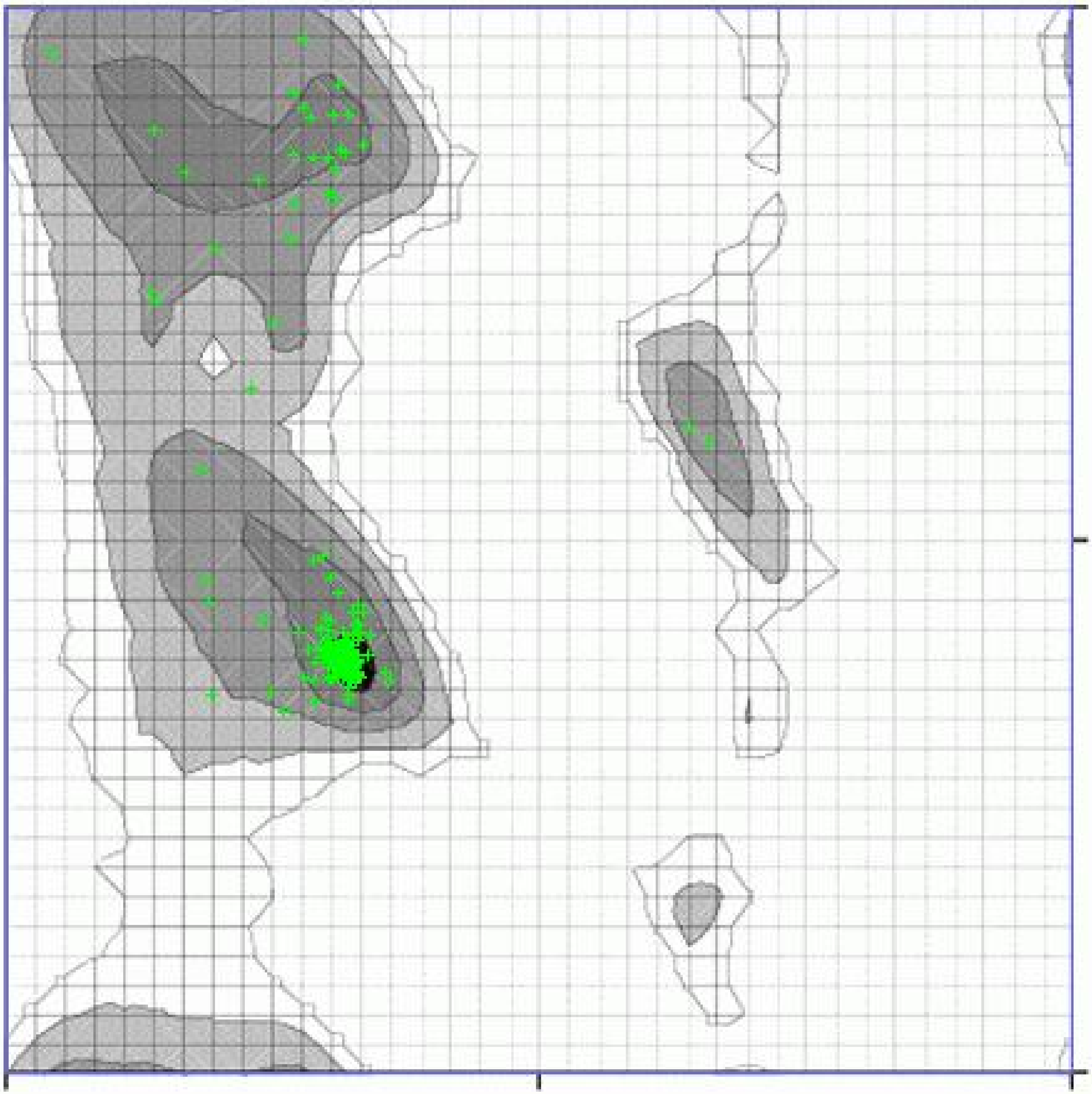

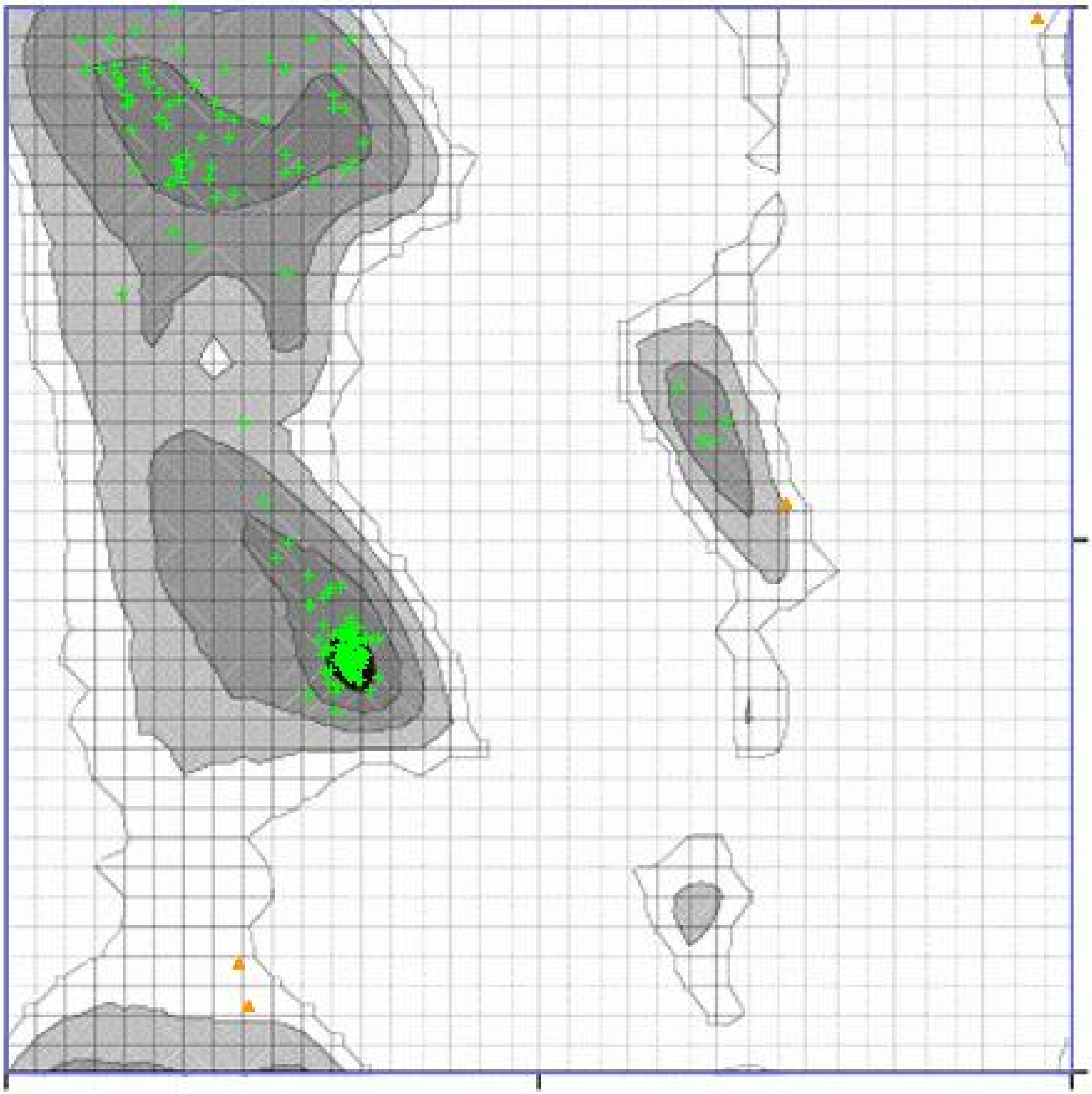

PROCHECK

Ramachandran Plot

saves

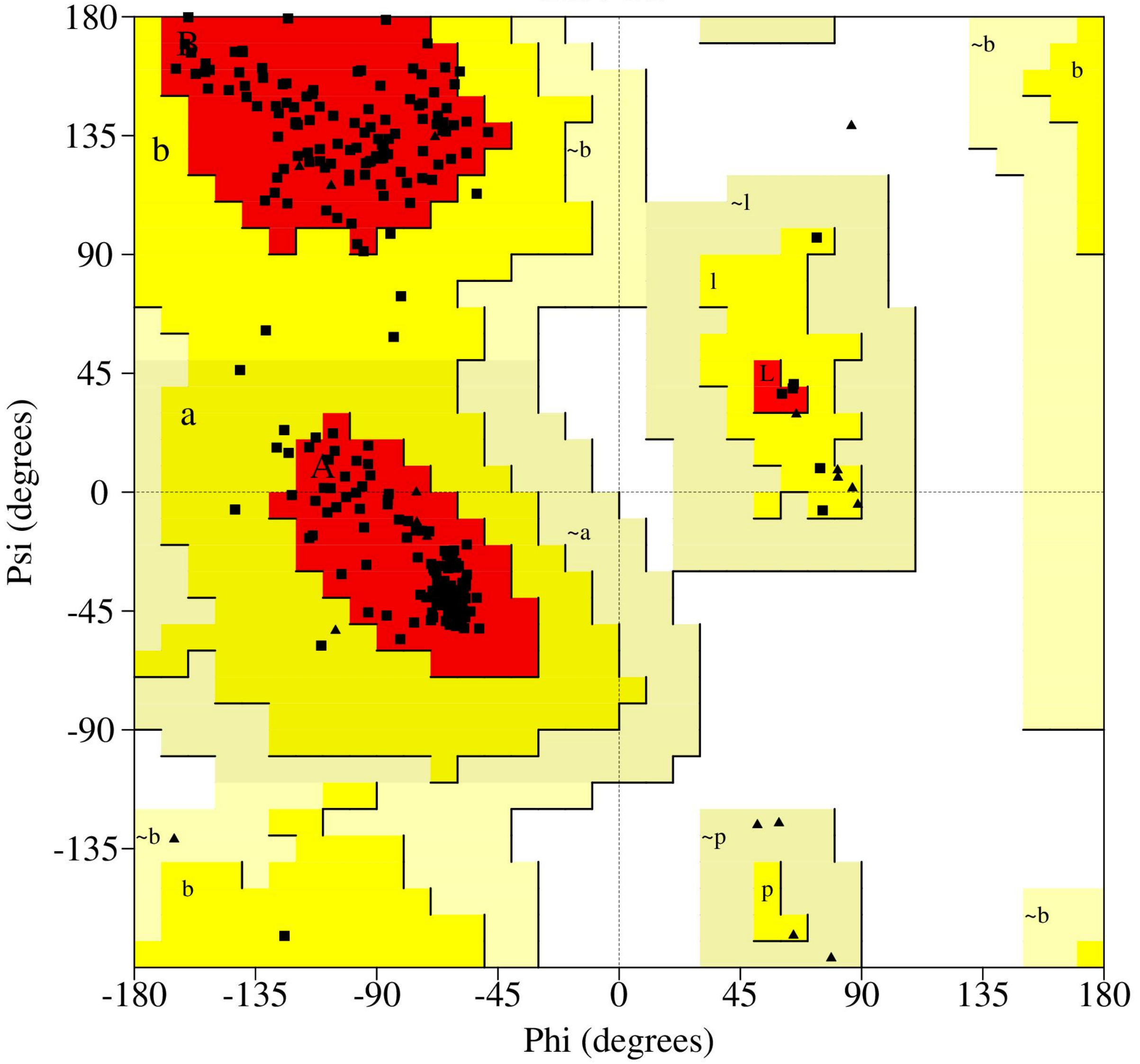

Plot statistics

Residues in most favoured regions [A,B,L]	252	93.7%
Residues in additional allowed regions [a,b,l,p]	17	6.3%
Residues in generously allowed regions [~a,~b,~l,~p]	0	0.0%
Residues in disallowed regions	0	0.0%
	----	------
Number of non-glycine and non-proline residues	269	100.0%
Number of end-residues (excl. Gly and Pro)	1	
Number of glycine residues (shown as triangles)	20	
Number of proline residues	13	

Total number of residues	303	

Based on an analysis of 118 structures of resolution of at least 2.0 Angstroms and R-factor no greater than 20%, a good quality model would be expected to have over 90% in the most favoured regions.

saves_01.ps

PROCHECK

Ramachandran Plot

saves

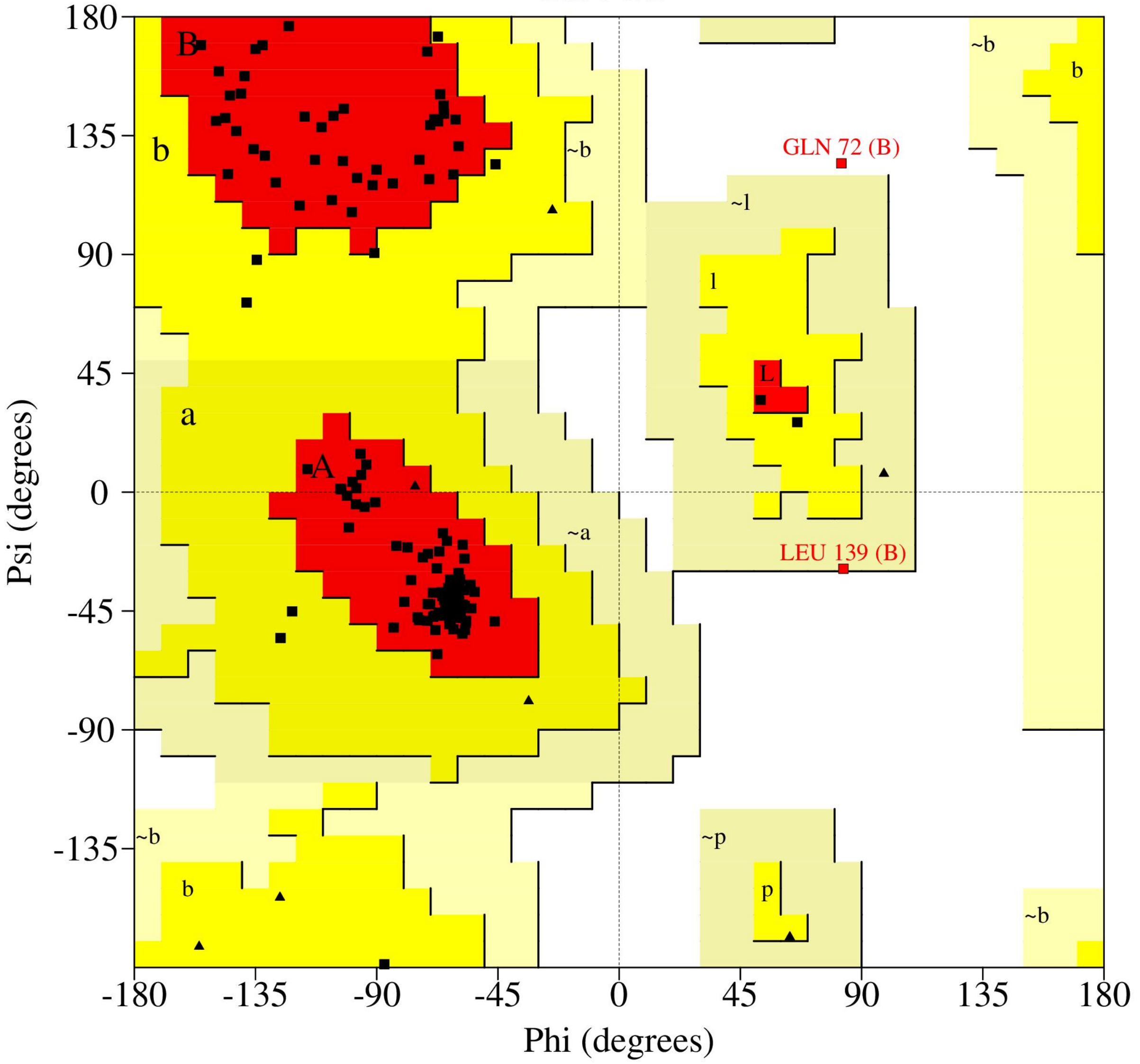

Plot statistics

Residues in most favoured regions [A,B,L]	140	94.0%
Residues in additional allowed regions [a,b,l,p]	7	4.7%
Residues in generously allowed regions [~a,~b,~l,~p]	1	0.7%
Residues in disallowed regions	1	0.7%
	----	------
Number of non-glycine and non-proline residues	149	100.0%
Number of end-residues (excl. Gly and Pro)	2	
Number of glycine residues (shown as triangles)	7	
Number of proline residues	7	

Total number of residues	165	

Based on an analysis of 118 structures of resolution of at least 2.0 Angstroms and R-factor no greater than 20%, a good quality model would be expected to have over 90% in the most favoured regions.
